# Modification of Cellulose Micro- and Nanomaterials to Improve Properties of Aliphatic Polyesters/Cellulose Composites: A Review

**DOI:** 10.3390/polym14071477

**Published:** 2022-04-05

**Authors:** Mariia Stepanova, Evgenia Korzhikova-Vlakh

**Affiliations:** Institute of Macromolecular Compounds, Russian Academy of Sciences, Bolshoy pr. 31, 199004 St. Petersburg, Russia; maristepanova@hq.macro.ru

**Keywords:** microcrystalline cellulose, nanocrystalline cellulose, cellulose fibers, cellulose modification, aliphatic polyesters, polyhydroxyalkanoates, poly(lactic acid), poly(ε-caprolactone), poly(glycolic acid), poly(lactic acid-co-glycolic acid), poly(hydroxybutyrate), poly(butylene succinate), (bio)composites, “green” materials, mechanical properties, thermal properties, degradation, biocompatibility

## Abstract

Aliphatic polyesters/cellulose composites have attracted a lot attention due to the perspectives of their application in biomedicine and the production of disposable materials, food packaging, etc. Both aliphatic polyesters and cellulose are biocompatible and biodegradable polymers, which makes them highly promising for the production of “green” composite materials. However, the main challenge in obtaining composites with favorable properties is the poor compatibility of these polymers. Unlike cellulose, which is very hydrophilic, aliphatic polyesters exhibit strong hydrophobic properties. In recent times, the modification of cellulose micro- and nanomaterials is widely considered as a tool to enhance interfacial biocompatibility with aliphatic polyesters and, consequently, improve the properties of composites. This review summarizes the main types and properties of cellulose micro- and nanomaterials as well as aliphatic polyesters used to produce composites with cellulose. In addition, the methods for noncovalent and covalent modification of cellulose materials with small molecules, polymers and nanoparticles have been comprehensively overviewed and discussed. Composite fabrication techniques, as well as the effect of cellulose modification on the mechanical and thermal properties, rate of degradation, and biological compatibility have been also analyzed.

## 1. Introduction

In recent decades, aliphatic polyesters have attracted enormous interest as an alternative to plastics derived from petroleum [1]. Aliphatic polyesters are biocompatible, biodegradable, and have an excellent ability to a number of processing techniques allowing the production of electrospun nanofibers, films, filaments, nonwoven materials, 3D-printed materials of different shapes, molded and pressed materials, nanocomposite bulk materials, etc. [2,3,4]. Degradation to nontoxic products, the possibility of recycling, thermoplasticity, nontoxicity, comparability of some parameters with poly(ethylene terephthalate) (PET) and polypropylene (PP) [5,6,7], low flammability, smoke and refractive index, and dyeability [8] are among other positive features of aliphatic polyesters. In sum, these advantages make aliphatic polyesters very attractive polymers for obtaining biomedical (drug-delivery systems, suture threads, scaffolds for tissue engineering, etc.) [5,9,10,11] and environmentally friendly materials (packaging and disposable items such as clothing, tableware, etc.) [8,12,13]. However, their high hydrophobicity, insufficient thermal stability, and mechanical and barrier properties limit their wide application for technical purposes. The most powerful way to modify the properties of aliphatic polyesters is to obtain various composites [14]. In this case, the properties of the matrix polymer can be adjusted by the selection of a certain filler. For example, metals [15], carbon nanotubes [16], graphene [17] and its derivatives [18], ceramics [19], and different organic nanoparticles [20,21,22] are considered to improve the properties of interest.

Despite the variety of potential fillers, the most attention is paid to micro- and nanomaterials that are nontoxic and inexpensive, which makes it possible to produce “green” biocomposites on an industrial scale. Cellulose micro- and nanomaterials are among the most potential fillers for producing such environmentally friendly and biocompatible composites [23,24,25,26]. Excellent mechanical properties, a large specific surface area of cellulosic materials, the possibility to obtain them from the wastes of various industries, as well as biodegradability and biocompatibility make their application as reinforcing materials for a variety of areas, including biomedicine and obtaining “green” materials, relevant. However, the hydrophilicity of cellulose impairs significantly its dispersion in hydrophobic polyesters, which leads to cellulose aggregation, poor dispersion in the matrix polyester, and as a consequence, unsatisfactory material properties [27,28]. This obstacle can be overcome by modifying cellulose materials to improve their compatibility with hydrophobic polymer matrices, and as a result, to provide a more homogeneous dispersion of the filler.

In recent years, much attention has been paid to improving the compatibility of cellulose with aliphatic polyesters by covalent and noncovalent modification [29,30,31,32,33,34,35,36,37,38]. Modification of the cellulose surface, in turn, affects the properties of the cellulose filler and allows the properties of aliphatic polyester/cellulose composites to be adjusted in a wide range. Recently, several review articles devoted to the composites based on poly(lactic acid) and cellulose [39,40] with special focusing on the processing techniques [41,42] and biofiber’s properties [43] have been published. Some reviews have partially discussed cellulose modification [39,44]; however, progress in this area has not been extensively overviewed.

In this comprehensive review, we have summarized the progress on the various approaches reported for the modifications of cellulose micro- and nanomaterials and the further preparation of composites with aliphatic polyesters. The different techniques such as adsorption, covalent modification with small molecules, grafting with polymers, and modification with inorganic and organic nanoparticles have been discussed. Unlike most reviews that consider only poly(lactic acid) (PLA), we have also included other aliphatic polyesters used to produce composites with modified cellulose, e.g., poly(glycolic acid), poly(ε-caprolactone), poly(hydroxybutyrate), poly(butylene succinate) and their copolymers. Furthermore, the effect of modification on various properties of composites, such as mechanical, thermal, degradation and biological ones, have been analyzed.

## 2. Cellulose Micro- and Nanomaterials

It is known that cellulose is the most abundant polysaccharide on our planet. Its main sources are primarily plants, including wood, as well as algae, tunicate, and metabolic products of some bacteria [45,46]. The highest cellulose content (more than 90%) is characteristic of “bacterial cellulose” (BC), while for other sources this value does not exceed 80% (plant—30–80%, tunicate—about 60%, algae—8–47%) [46,47,48,49]. The exception is mature cotton fibers, which consist almost entirely of cellulose (88.0–96.5%) [50]. Accordingly, BC and mature cotton are characterized by fewer impurities, such as lignin and hemicellulose, which are present in large amounts in plant and algae cellulose [46,50]. Another feature of BC is the presence of a finer mesh structure [46]. Furthermore, the degree of crystallinity for cellulose from different sources also varies quite a lot. Regardless of the source, cellulose is a linear homopolysaccharide and consists of β-1,4-glycosidic bonded anhydro-D-glucose units [49,51]. A large number of Van der Waals and hydrogen interactions are formed between and within the polymeric cellulose chains, which lead to the formation of three-dimensional hierarchical structures, the structural unit of which is an elementary fibril [52]. Elementary fibrils, also called elementary nanofibrils, are threadlike bundles of cellulose molecules consisting of alternating crystalline and a number of amorphous domains providing fiber flexibility [26]. Elementary fibrils due to aggregation are packed into microfibrils, which, in turn also aggregate, and this leads to the formation of cellulose fiber [53]. The widths of elementary fibrils and microfibrils range from 1.5 to 5 nm [54,55] and from 10 to 30 nm [49,54], respectively, and the width and length of microfibril aggregates can reach the order of 100 nm and tens of micrometers [49,51,53,55], respectively. The large number of hydroxyl groups (three reactive groups in each monomeric unit) and the supramolecular structure of cellulose determine its physical and chemical properties (insolubility in water and basic solvents, semicrystallinity, good mechanical properties, relative reactivity) [26,55,56,57].

**Figure 1:**
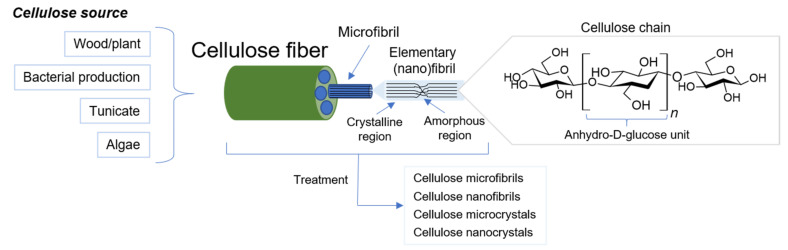
Cellulose from source to molecule and micro- and nanomaterials.

Depending on the origin and the method of isolation, the degree of polymerization (DP) of cellulose and the molecular orientation of its chains can be different. For native cellulose, the most common crystalline structure is cellulose I, which under the influence of sodium-hydroxide solution or recrystallization changes to the most stable crystalline state, cellulose II. More details about the different forms of cellulose can be found elsewhere [49,51,55,58,59]. The degree of polymerization of cellulose ranges from a few hundred to several tens of thousands [46,49,58]. Given the structure of cellulose, cellulose objects can be produced as fibers, micro/nanofibrils, and micro/nanocrystals [26,44], which vary in degree of polymerization, crystallinity, and shape [60]. Figure 1 schematically demonstrates the general hierarchical structure and structure of a single polymer chain of cellulose with a list of the main cellulose micro- and nanomaterials obtained.

**Figure 2:**
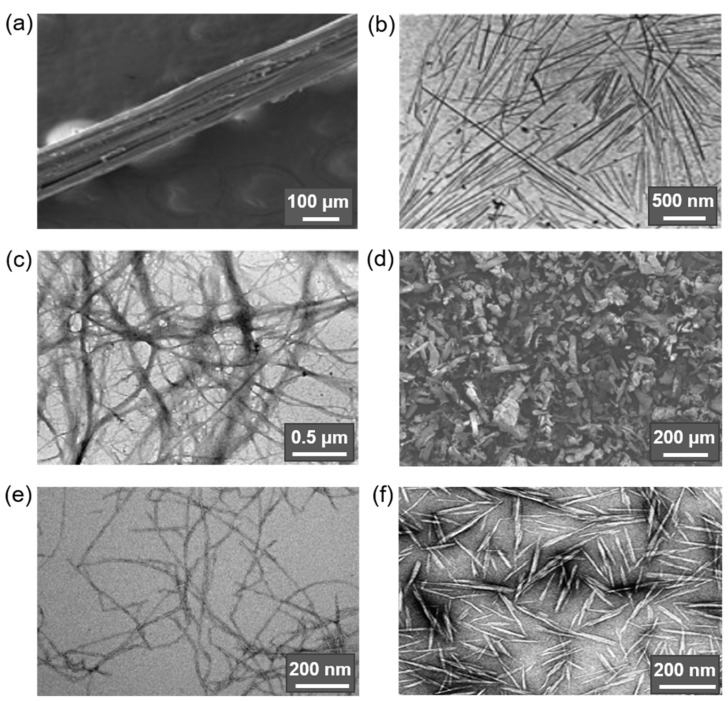
Overview of cellulose micro- and nanomaterials commonly used as fillers to prepare composite materials. Electron micrographs of (**a**) sisal fiber (scanning electron microscopy (SEM), reproduced from [62] under the terms of the Creative Commons CC BY license), (**b**) tunicate whiskers (transmission electron microscopy (TEM), reproduced from [63] with permission of American Chemical Society), (**c**) sugar beet CMF (TEM, reproduced from [64] with permission of Elsevier), (**d**) CMC, commercial (SEM, reproduced from [65] with permission of John Wiley & Sons, Inc), (**e**) wood CNF (TEM, reproduced from [66] with permission of American Chemical Society), (**f**) CNC from ramie fibers (TEM, reproduced from [67] under the terms of the Creative Commons CC BY license).

The nomenclature used to designate the various types of micro- and nanocellulose materials is currently ambiguous. Thus, cellulose nanocrystals (CNC) are called nanocrystalline cellulose (NCC), cellulose nanowhiskers (CNW), cellulose whiskers, nanocrystals, nanofibers, nanoparticles, nanorods, rod-like cellulose crystals, cellulose microcrystals (CMC), cellulose microcrystallites, cellulose microfibrils (CMF) [26,39,51,55]; cellulose microfibrils (CMF) are called microfibrillated cellulose (MFC), microfibrillar cellulose, nanofibrillated cellulose (NFC), cellulose nanofibrils (CNF) [26,55]; a synonym of microcrystalline cellulose (MCC) is whiskers [53]. Some time ago, the Technical Association of the Pulp and Paper Industry proposed to standardize the terminology used (nomenclature and abbreviation). According to the recommendations (WI 3021), depending on the dimensions (width (w) and length/width ratio (L/w)), cellulose materials are divided into: cellulose nanocrystals (CNC, w = 3–10 nm, L/w > 5), cellulose nanofibrils (CNF, w = 5–30 nm, L/w > 50), cellulose microcrystals (CMC, w = 10–15 μm, L/w ˂ 2), cellulose microfibrils (CMF, w = 10–100 nm, L/w > 50) [61]. The main cellulose types found in the literature and used in the production of composite materials are shown in Figure 2.

The size, type, and consequently the physical and chemical properties of the resulting cellulose materials are influenced by the source of origin, processing, and extraction method [44]. For instance, the use of mechanical action alone or its combination with chemical treatment of previously purified cellulose pulp/fibers (e.g., carboxymethylation or TEMPO-mediated oxidation) and/or enzymatic hydrolysis results in thin long flexible micro- (CMF) or nanofibrillar (CNF) structures with alternating crystalline and noncrystalline domains. In turn, acidic hydrolysis produces stiffer particles with a high degree of crystallinity (CMC and CNC), which are the result of the action of acid on both amorphous and crystalline domains. Thus, in the first case, the obtained cellulose micro- and nanofibrils retain the inherent semicrystallinity and high aspect ratio (L/w, over 50 for CMF and CNF) [39,46,49,55], while acid exposure reduces the number of defects in the structure and results in more highly crystalline materials with much lower L/w values (8 to 67 for CMC and CNC) [26,53,68].

Despite the existing terminology recommendations for cellulosic micro- and nanomaterials (see above), the use of terminology in the practice of current publications varies. Nevertheless, we have attempted to generalize the available data on the size of the various cellulose-based materials used. The preparation methods and summarized descriptions and characteristics of the obtained micro- and nanocellulose materials found in literature are presented in Table 1 [26,27,28,39,44,46,69,70].

The degree of crystallinity and DP for all obtained materials depends largely on the source of the cellulose as well as the processing technique. The found values are very scattered. For example, DPs for micro- and nanoobjects in the literature range from 100 to 15,000 [46,70], and the degrees of crystallinity vary from a few dozen to more than 90% [26,27,55,70]. For instance, the degree of crystallinity for BC and tunicin (cellulose from tunicate) is 80–100%, for cellulose from algae it is more than 70%, and cellulose from plants it is 40–60% [39,46].

The source of cellulose also has a significant impact on its mechanical properties. Young’s tensile modulus for cellulose fibers varies from 5 to 200 GPa [39,46,71]. The highest values from this range are typical for tunicin fibers (from 110 GPa), while for fibers from other origins the elastic modulus does not exceed 115–130 GPa [39,46]. Elongation at break and tensile strength of cellulose fibers are in the ranges of 1–30% and 0.2–1.2 GPa, respectively [26,71]. The application of cellulose-fiber treatments that help to reduce the amorphous components in the chain packing thereby leads to a decrease in DP, an increase in crystallinity and, as a result, an increase in the mechanical properties of the resulting cellulose material compared to the original fibers [39,55]. The theoretically calculated Young’s modulus (E) of an ideal cellulose crystal (along the axis of the cellulose chain) is 167.5 GPa [72]. According to the published data, the practically identified Young’s modulus values for micro/nanocrystals range from 60 to 220 GPa [26,39,44,46,49,73]; for micro/nanofibers from 14 to 84 GPa [39,46,73]; and for a single tunicin microfibril, a value of about 150 GPa has been found [74]. The established tensile-strength data are in the range of 1–10 GPa for CMC and CNC [39,44,46,58]; about 2–6 GPa for cellulose nanofibers [66]; and about 4–8% elongation at break for wood-cellulose CNF has been reported [46]. The above data indicate the excellent mechanical properties of these micro- and nanoscale cellulose materials. The characteristics of crystalline cellulose (density 1.5–1.6 g/cm^3^) are close to—and in some cases significantly higher than—those of glass fibers used for composites (E about 70 GPa, density 2.6 g/cm^3^), Kevlar (E 60–125 GPa, density 1.45 g/cm^3^) and steel (E 200–220 GPa, density about 8 g/cm^3^) [61].

**Figure 3:**
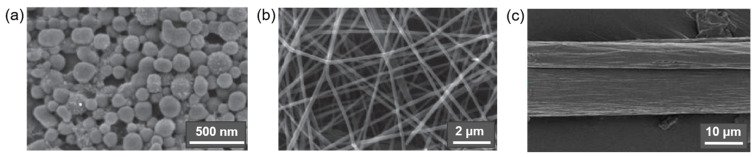
SEM images of (**a**) ANC from CMC (reproduced from [76] under the terms of the Creative Commons CC BY license), (**b**) CNY from carboxymethyl cellulose sodium salt with polyethylene oxide at ratio 1:1 (reproduced from [77] with permission of John Wiley & Sons), (**c**) cellulose filament from carboxymethylated CNF (reproduced from [78] under the terms of the Creative Commons CC BY license).

In addition to the above subgroups, cellulose materials such as amorphous nanocellulose (ANC) and cellulose nanoyarn (CNY) are also mentioned in the literature. ANC are obtained from regenerated cellulose by acid hydrolysis and ultrasonic treatment and are generally elliptical particles 50–200 nm wide with DP of 60–70 with an amorphous structure and hence poor mechanical properties. CNYs are electrospun nanofibers; their width and DP range from 500–800 nm and 300–600 nm, respectively [26]. CNFs can be made into cellulose filaments by flow-focusing, wet-extrusion, or spinning processes, but their mechanical properties are also inferior to the more highly crystalline forms of micro- and nanocellulose materials [46,75]. Figure 3 shows these three forms of cellulosic materials.

## 3. Aliphatic Polyesters

**Figure 4:**
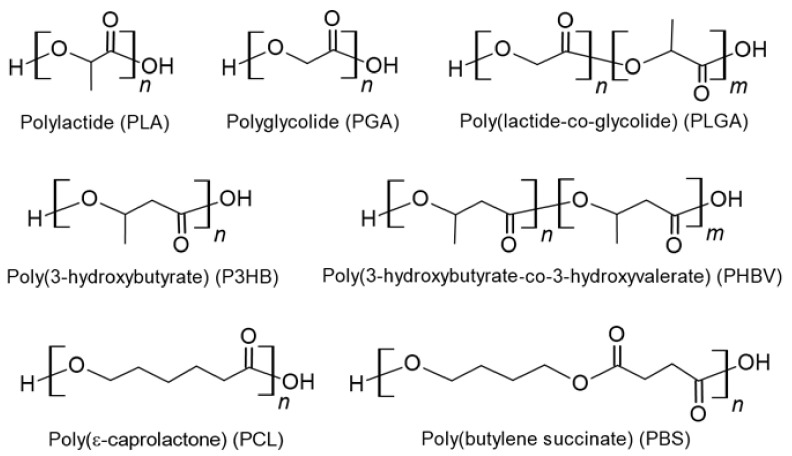
Structures of PLA, PGA, PLGA, P3HB, PHBV, PCL, and PBS.

Aliphatic polyesters (polyhydroxyalkanoates) are biocompatible and biodegradable materials. Currently, they are of interest for the fabrication of biomedical and environmentally friendly materials to replace petroleum-based plastics. Polylactide, polyglycolide, polyhydroxybutyrate, polycaprolactone, poly(butylene succinate) and some copolymers based on them are among the widely considered aliphatic polyesters [79,80,81,82,83]. The chemical structures of key aliphatic polyesters are illustrated in Figure 4, while their key characteristics are summarized in Table 2.

### 3.1. Poly(lactic acid)

Poly(lactic acid) (PLA) or polylactide is a synthetic, thermoplastic, biocompatible, and biodegradable aliphatic polyester derived from renewable lactic acid [7,11,84]. PLA can be produced by lactic-acid condensation or by the ring-opening polymerization (ROP) of lactide (lactic-acid cyclic dimer) [5,7,11,14,44]. Polycondensation of lactic acid allows the synthesis of only a low-molecular-weight polymer due to the side reaction of hydrolysis preventing the production of high-molecular-weight polymer chains. In contrast to polycondensation, ROP provides PLA with high molecular weight but requires the use of catalysts [7]. A combination of these methods is commonly used to produce PLA on an industrial scale [3]. In this case, a low-molecular-weight polymer is synthesized from lactic acid (2-hydroxypropionic acid) by polycondensation, then the formed polymer is depolymerized to form lactide, which is further used to produce PLA of high molecular weights by ROP [3,7].

The presence of optical isomers of lactic acid and lactide (L-lactic acid, D-lactic acid, L,L-lactide or L-lactide, D,D-lactide or D-lactide, D,L-lactide or meso-lactide) leads to obtaining PLA of four types: isotactic and optically active poly-L-lactide (PLLA) and poly-D-lactide (PDLA), syndiotactic and atactic optically inactive poly-D,L-lactide (PDLLA) [44,84,85]. Equimolar racemic mixture of L- and D-enantiomers of lactide (rac-lactide) is also designated as D,L-lactide [7,85]. Chemical structures of stereoisomers of monomers and PLA can be found elsewhere [44].

The molecular structure (enantiopure) of PLA and heat treatment affect its crystallinity [3,84]. Optically inactive PDLLA is amorphous, whereas stereoregular PLLA and PDLA are capable of homocrystallization [84], forming α- (highest thermodynamic stability), β- or γ-crystalline forms depending on composition [3,6]. Blending PLLA and PDLA leads to their cocrystallization and the formation of a stereocomplex with a different crystal structure, characterized by an increase in melting temperature (*T_m_*) by about 50 °C relative to the homopolymer PLLA or PDLA [6,101]. The parameters of the crystalline forms of PLA can be found in detail elsewhere [6]. Stereochemistry has a tremendous influence on the supramolecular structure of PLA. The presence of more than 10 mol% of the links in the polymer chain different from the basic optical form leads to a significant decrease in crystallinity [84,102]. Branching of the polymer chain also leads to a rather significant decrease in the crystallinity and glass-transition temperature (*T_g_*) of PLA [102]. The crystallinity of PLA is also affected by the molecular weight of the polymer, various treatments, the introduction of nucleation agents, plasticizers into the matrix, and for final products, PLA crystallization can be initiated by temperature annealing [3,6,103].

Crystallinity, as well as the parameters listed above, determine the physical (thermal, rheological, barrier, etc.) and mechanical properties, as well as the degradability of PLA [3,7,44,104,105]. The high degree of crystallinity of polylactide leads to excellent thermal and mechanical properties [44]. However, a high degree of crystallinity is not always necessary and is determined by the application of the final polymer product. For example, the rapid crystallization can complicate stretching the product by blow molding, can diminish the optical transparency of the product, such as a bottle, and can increase the degradation time of the polymer, which may limit its use for some biomedical applications. At the same time, the presence of thermal stability due to high crystallinity is very important for products formed by injection molding [44]. It has been reported that the increase in molecular weight and crystallinity is accompanied by an increase in viscosity and softening point, so that the behavior of PLA in the melt becomes similar to polystyrene [85]. The thermal stability of PLA is similar to poly(vinyl chloride) (PVC), but significantly inferior to PP, polyethylene (PE), polystyrene (PS), and PET [85].

The glass-transition temperature and melting point of PLA are important parameters for predicting the material properties [84]. Both are influenced by the molecular weight of the polymer. *T_g_* and *T_m_* increase sharply when the number-average molecular weight (*M_n_*) increases to 80,000 and 100,000, respectively, and then remain almost unchanged [102]. As optical purity decreases with a constant molecular weight, a decrease in glass-transition temperature is observed. Moreover, PDLA is characterized by lower *T_g_* values than PLLA with the same molecular weight [3,102]. In turn, *T_m_* is more influenced by the amorphous state of PLA than *T_g_*, due to the lack or complete absence of a crystalline phase [102].

The commercially available type of PLA produced on a large scale is mainly PLLA [6,84], since about 90% of all lactic acid is produced from renewable sources by microorganisms as L-isomer [84]. Thus, a commercial polylactide is a semicrystalline polymer with a *T_g_* of 55 to 65 °C and a *T_m_* of 140 to 180 °C depending on the amount of the D-enantiomer impurity [94]. In comparison with petrochemical-based plastics, PLA has a slow crystallization rate, low impact strength, low thermal resistance, and low glass-transition temperature and fragility [11,94]. For instance, to consider the substitution of PET by PLA for packaging fabrication, the barrier properties of PLA need to be improved. Typically, aliphatic polyesters with molecular weights greater than 60,000 are used for packaging, agricultural, and biomedical applications [11].

### 3.2. Poly(glycolic acid)

Poly(glycolic acid) or polyglycolide (PGA) and its copolymer with PLA (PLGA) is among the most widely studied and used polymers [84,86]. PGA is a semicrystalline, biodegradable, biocompatible aliphatic polyester that differs from PLA in the absence of a methyl group in the monomer unit (glycolic acid residue) [86,93]. PGA can be obtained by polycondensation of glycolic acid (difficult to obtain high molecular weights), ROP of glycolide (more economical, but pure monomer is required), and solid-phase polycondensation of halogen acetates (low degree of polymerization) [86]. The synthesis conditions determine PGA molecular weight, crystallinity, *T_m_* and *T_g_*, and terminal groups. The growth of the molecular weight of PGA contributes to an increase in crystallinity, mechanical properties and a decrease in the biodegradation rate. Acceptable mechanical properties of PGA are achieved at molecular weights greater than 30,000 [86]. Due to the high degradation rate of PGA, its synthesis is more difficult and expensive than for PLA.

Mechanical, thermal, degradation properties and density of PGA are determined by molecular weights, dispersity, and crystallinity. PGA is characterized by high crystallinity. The most common crystallization degree is 45–55%, but 77% has also been reported. Due to the stabilized crystal cage, PGA has a high melting point (220–230 °C) and poor solubility (soluble only in highly fluorinated solvents such as hexafluoroisopropanol) [86]. The glass-transition temperature (*T_g_*) of PGA is higher than the ambient temperature, but close to human body temperature (35–40 °C), which makes the material elastic when introduced into the human body (e.g., implantation) [86,93]. PGA is characterized by poor thermal stability because *T_m_* is very high and close to the degradation temperature [90,92]. The lack of solubility in conventional organic solvents and the narrow processing window of PGA melt create a problem in obtaining products based on it [93]. At the same time, the supramolecular structure of PGA provides excellent mechanical properties [86,93]. For example, the elastic modulus (*E*) of PGA is higher than that of other synthetic biodegradable polymers (PLLA, PDLLA, poly-ε-caprolactone) and is 6–7 GPa [86,93]. The high density of PGA (1.50–1.71 g/cm^3^), due to the molecular-packing features, provides high gas-tight properties of the polymer, exceeding this parameter of polyethylene terephthalate (PET) by 100 times [86,92].

Currently, PGA is of great interest for renewable industry and biomedical applications due to its thermal properties, biocompatibility, biodegradation, nontoxicity, excellent mechanical characteristics, and low gas permeability. The obstacles of PGA application are overcome by making PGA-based copolymers and composites [86,93,106]. For example, by copolymerizing glycolide and various enantiomers of lactide and varying their ratios, the properties of the resulting PLGA (stiffness, crystallinity, melting point, and biodegradation rate) can be set. For example, PLGA demonstrates mechanical properties similar to those of human calcareous bone. In addition, PLGA is widely used as implants, micro- and nanoparticles for drug delivery [107,108].

### 3.3. Poly(hydroxybutyrate)

Poly(3-hydroxybutyrate) (P3HB, PHB) is a thermoplastic, biodegradable, semicrystalline, linear microbial aliphatic polyester [5,8,79,87]. Biosynthesis in cells of natural/transgenic plants and bacterial fermentation, including the use of genetically modified microorganisms, are the main ways for the production of P3HB [8,79,87]. In particular, P3HB is produced by prokaryotic microorganisms from sugar-based media (agricultural industrial wastes, hydrolysates of some wood) and other carbon sources in the form of inclusion bodies, which serve as intracellular bacterial depots storing carbon and energy [5,79,87]. Microorganisms may accumulate up to 40–50% of P3HB from the dry-cell mass, and in the case of *Alcaligenes eutrophus* the accumulation may reach up to 96% of the dry cell mass [87]. Depending on the conditions and isolation forms, the resulting P3HB can have different characteristics (molecular weight, crystallinity, mechanical properties and ability to biodegrade) [8,79].

P3HB exhibits optical activity due to a chiral central carbon, and the main natural configuration is poly(D-3-hydroxybutyrate) [87]. The stereostructure and tacticity of P3HB can be specified by chemical synthesis, obtaining isotactic, syndiotactic, or atactic PHB [8,88,109]. The number of monomeric units in P3HB can vary in different range: (1) over than 10,000 for P3HB produced as cytosolic inclusions of bacteria, (2) 100–300 for P3HB from cell membranes and (3) up to 30 monomeric units for P3HB for other natural sources, including human tissues [8]. P3HB with a number of monomeric units greater than 1000 (molecular weight greater than 100,000) can be obtained chemically from β-butyrolactone [8,109].

The linear structure of the P3HB chain ensures its high crystallinity with the presence of an amorphous phase in addition to the crystalline phase [79]. The crystallinity of P3HB can vary in a wide range from 50 to 80%, and as with PLA, has a significant effect on the mechanical properties [79,87]. P3HB is generally characterized as a strong and stiff polymer with low thermal stability and low crystallization rate. Secondary crystallization of P3HB occurs at room temperature with the formation of amorphous lamellae, leading to polymer brittleness [87,110]. P3HB has piezoelectric properties and is also characterized by good resistance to acids, bases, and ultraviolet radiation [87]. In addition, P3HB has better barrier properties than PP, PE, PVC, and PET, and is characterized by some other properties similar to or superior to those of PP and PE [79]. In addition to these advantages, P3HB is a biocompatible and nontoxic polyester, which makes it a promising environmentally friendly alternative to petrochemical polymers, and also demonstrates suitability for various tissue-engineering and other biomedical applications (scaffolds, surgical threads, drug-delivery systems, surgical mesh, etc.).

The low thermal stability is due to the close values of the melting and degradation temperatures of P3HB, which leads to a narrow heat-treatment window. Thus, thermal degradation of the polymer melt can occur during processing [110,111]. This problem can be partially solved by using a random copolymer of 3-hydroxybutyric and 3-hydroxyvaleric acids (poly(3-hydroxybutyrate-co-3-hydroxyvalerate) or poly(3-hydroxybutyric acid-co-3-hydroxyvaleric acid) abbreviated as PHBV or PHBHV. PHBV as well as P3HB homopolymer is characterized by high crystallinity, biocompatibility, biodegradability in different environments, good barrier properties, nontoxicity, UV stability, similarity to P3HB in solubility and chemical stability, hydrophobicity, and low impact resistance and fragility. Unlike P3HB, PHBV has a lower *T_m_*, higher surface tension and flexibility [111]. Thus, PHBV appears to be technologically more attractive and of interest as materials for biomedical applications, agriculture, and packaging materials, and has been developed on an industrial scale [88]. PHBV copolymer can be produced by various microorganisms [111], including recombinant strains, in amounts up to 80% of dry-cell weight, and PHBV composition can vary over a wide range depending on substrate composition [111].

In addition to PHBV, lower melting-point values are observed for another copolymer, namely poly(3-hydroxybutyrate-co-4-hydroxybutyrate) (P(3HB-co-4HB)). Thus, an increase in the number of 4-hydroxybutyrate units (from 0 to 38 mol%) provides a significant decrease in *T_m_* (from 176 to 54 °C), and with a further increase in the proportion of 4HB the melting temperature of copolymers practically does not change. P(3HB-co-4HB) is a thermoplastic biodegradable aliphatic polyester produced by bacterial fermentation. The ratio of monomeric units is largely determined by the substrate used. Moreover, during biosynthesis the production of a mixture of copolymer compositions with a wide range of monomer compositions is observed, including the presence of P4HB, which significantly affects the characteristics of isolated polymers [112]. Regarding P3HB, P4HB is a relatively new material, which can also be obtained by polycondensation of 4-hydroxybutyric acid or ROP γ-butyrolactone. P4HB is also nontoxic, biocompatible, biodegradable, UV-resistant, demonstrates relatively good barrier properties, and exhibits optical activity, and therefore can be used for biomedical and packaging applications [79].

### 3.4. Poly(ε-caprolactone)

Poly-ε-caprolactone (also called, polycaprolactone, PCL) is a biodegradable, hydrophobic, semicrystalline synthetic aliphatic polyester whose monomer unit is built from 6-hydroxyhexanoic acid (6-hydroxycaproic acid) [89,98]. PCL can be synthesized by polycondensation of 6-hydroxyhexanoic acid, but because of the equilibrium nature of this process and the need to remove the water formed during the reaction, obtaining a polymer with a high degree of polymerization and molecular weight values above 10,000 is challenging [90]. Thus, the most promising method is the ROP of ε-caprolactone, which allows the production of PCL with low dispersity and high molecular weights. However, this method of synthesis requires the use of catalysts, often based on metals, which can possess toxic effects [89]. The ε-caprolactone monomer may be obtained by oxidation of cyclohexanol by peracetic acid (industrial method of production) [11], and also by microorganisms as one of the intermediate products [89]. PCL can be synthesized in a wide range of molecular weights. The crystallinity degree of PCL can be up to 69%, and it decreases with the growth in molecular weight [89,98]. It was reported that the nonenzymatic hydrolytic degradation process increased the crystallinity of the PCL sample from the initial 45% to nearly 80% [113]. As with other crystalline aliphatic polyesters, the molecular weight and corresponding degree of crystallinity of PCL have a significant impact on its physical, mechanical, thermal, and degradation properties [89,98]. For example, PCL with *M_n_* below 15,000 has a low viscosity and forms very brittle materials, while polymer with *M_n_* from 25,000 to 90,000 is characterized by desirable mechanical and rheological properties [90]. A rapid thermal degradation is observed for PCL at temperatures above 170 °C, but its low melting (55 to 70 °C) and glass-transition temperatures (−65 to −54 °C) allow its easy processing, which distinguishes PCL from other biodegradable aliphatic polyesters with higher *T_m_*, such as PLA, PGA, their copolymers, PHB and its copolymers [79,90]. In addition to higher thermal stability, PCL in comparison with its biodegradable analogues is characterized by higher viscoelastic properties [98]. Thus, the listed characteristics along with variable viscosity make PCL very technological, suitable, and promising for melt processing such as melt extrusion, electrospinning, injection molding, and 3D printing [90].

PCL is strongly inferior in its mechanical properties to other aliphatic polyesters, and the materials made from it—depending on the architecture, and especially porous ones—are characterized by an even lower load-bearing capacity. This disadvantage imposes significant limitations on the use of PCL materials. This problem can be solved by obtaining copolymers with PCL or composites based on it [90,98]. It should be noted that PCL can be blended with many polymers (PVC, polycarbonates, PLA, PLGA, and several others to form mechanically compatible composites. This property of PCL opens up many opportunities to regulate mechanical, biodegradation, and biological properties for a variety of tasks [89,90,98]. Finally, in comparison to PLA, the low degradation rate of PCL also contributes to the minimal formation of physiological problems caused by pH shifts in the environment during the biodegradation of PCL [90,98].

### 3.5. Poly(butylene succinate)

Poly(butylene succinate) (PBS) is a synthetic, biocompatible, semicrystalline, thermoplastic, biodegradable aliphatic polyester [80,91]. PBS is prepared by the polycondensation of succinic acid (or dimethylsuccinate) with 1,4-butanediol. The monomers (succinic acid and 1,4-butanediol) can be obtained from renewable or fossil-based resources [91]. Safe and accessible microwave radiation can be used to synthesize PBS, resulting in reduced reaction times and increased yields. PBS can be synthesized with high molecular weights and is a commercial product, but its cost is higher compared to common petrochemical plastics. PBS is characterized by two types of crystalline structures (α- and β-form). A β-crystalline structure is observed for the material in the state of deformation [80]. The degree of crystallinity for PBS is 35–45% [91]. Mechanical and thermal properties, as well as biodegradation, depend on the molecular weight of the polymer and its crystallinity [80,91]. PBS is characterized by flexibility and tensile strength close to that of PE and PP [91]. Some physical properties of PBS are comparable to PET. PBS is characterized by good thermal properties. The glass-transition temperature of this polymer is considerably lower than room temperature and ranges from −45 to −10 °C. The melting point of PBS is higher than for PCL but lower in comparison with PLA, PHB, PHBV, and PGA, and varies in the range of 90–120 °C. In view of the above, for PBS various methods of processing are applicable: extrusion, thermoforming, injection molding, etc. These properties, along with biodegradation, distinguish PBS from polyolefins [91]. However, PBS is characterized by disadvantages such as low melt viscosity, slow crystallization rate, gas tightness, and relative brittleness [80,91]. To improve these characteristics, as well as to vary the rate of decomposition and reduce the cost of materials, it is possible to obtain copolymers and composites based on PBS [91]. Furthermore, the introduction of plasticizers in the PBS matrix can improve the rheological properties of this polymer and reduce brittleness [80]. Biodegradability, environmental friendliness, chemical resistance, transparency, physical and mechanical properties, recyclability, and processability allow its application in various fields, first of all in packaging and disposable tableware, but also in textiles, automotive industry, agriculture, forestry, fisheries, medicine, etc.

## 4. Modification of Cellulose Micro- and Nanomaterials

### 4.1. Adsorption

Physical modification, or adsorption, is one of the simplest and oldest techniques to modify cellulose nanomaterials [114]. To date, there are a lot of publications describing the modification of cellulose nanomaterials with small molecules and polymers. Selected examples are summarized in Table 3. Basically, the modifying agents are surface-active molecules, or surfactants. Being amphiphilic, they serve as intermediates between hydrophilic cellulose and hydrophobic polyesters. The hydrophilic fragments of surfactants interact with cellulose hydroxyls, while other parts of the (macro)molecule surround the surface like “brushes”, preventing the direct interaction between cellulose fibers/particles in nonpolar surroundings.

Among the small molecules, ethoxylated nonylphenol phosphate ester and cetyltrimethylammonium bromide (CTAB) are the most widely used compounds. Li et al. showed that the modification of CNC with low-molecular surfactants did not affect the size of nanocrystals and their distribution [115]. As for modification with polymeric surfactants, such polymers as lignin, poly(vinyl alcohol) (PVA), poly(vinyl acetate) (PVAc), poly(N-vinylpyrrolidone) (PVP), and poly(ethylene glycol) (PEG) are widely applied to enhance the compatibility of cellulose nanomaterials with aliphatic polyesters.

The most common technique for the physical modification of cellulose is the adding of surfactants to aqueous cellulose dispersion, followed by freeze-drying [116,117,118]. The success of modification of cellulose via adsorption was testified by several groups by such methods for structure characterization as Fourier transform infrared (FTIR) spectroscopy [115,118,119,120] and solid-state ^13^C NMR spectroscopy [116]. Besides these, a decrease in turbidity also can indirectly testify the surface modification. In particular, Gois et al. also evaluated the turbidity of the dispersions of the neat CNC and CNC modified with different PEGs or Pluronic VR L44 in chloroform [117]. The authors found that pure CNC began to precipitate after 2 min, while the modified CNC started to reduce its turbidity only after 3–5 min, depending on surfactant. 

**Table 3:** Selected studies on modification of cellulose micro- and nanomaterials via adsorption of small molecules and polymers.

Type of Cellulose	Modifier	Filler Content (wt%)	Matrix Aliphatic Polyester	Processing/ Design of Composites	Characterization Methods	Refs.
CNC	Ethoxylated nonylphenol phosphate ester	5	PLA/PHB	Melt blending/ Films	TEM, FTIR, XRD, TGA and DSC	[119]
CNW	Ethoxylated nonylphenol phosphate ester	5	PLA	Extrusion + Hot pressing/ Strips	GPC, SEM, TEM, DMA and tensile tests	[114]
CNC	Ethoxylated nonylphenol phosphate ester	1 or 5	PLA/Ag NPs	Casting/Films; Electrospinning/Mats	TEM, FE-SEM, AFM, DSC, DMTA and tensile tests	[121,122]
CNC	CTAB	0.5, 1, 3 or 5	PLA	Hot pressing/Strips	FTIR, UVis, TEM, SEM, TGA, DTG and tensile tests	[115]
CNC	CTAB	1–3	PLA/rGO	Hot pressing/Sheets	FTIR, XRD, AFM, FE-SEM, TGA, DTG, WVP, WAXD, mechanical and MTT-tests	[118]
CNC	Decamethylene dicarboxylic dibenzoyl hydrazide	1 or 3	PLLA	Torque rheometry or Casting/Films	FTIR, XPS, AFM, SEM, TGA, WAXD, DSC, DMA and tensile tests	[123]
Cellulose fibers	Dopamine	40	PLA	Extrusion + hotmolding	FTIR, SEM, XRD, DSC, TGA and mechanical tests	[124]
CNF	Lignin	1, 3 or 5	PLA	Extrusion/Filaments	FTIR, SEM, DSC, DMA, tensile tests	[125]
CNC	Lignin	0.3–2.5	PLA	Compression molding/Disks	Optical microscopy, SEM, DSC, DMA and rheological tests	[104]
Cellulose fibers	Lignin and tannin	35	PLA	Compression molding/Sheets	NMR, FTIR, SEM, TGA, DMA, water sorption, SBS and flexural tests	[116]
CNC	Poly(vinyl alcohol)	1	PLA, PLA/PEG	Casting/Films	ATR-FTIR, XRD, TGA, DSC, mechanical tests	[126]
CNC	Poly(N-vinylpyrrolidone)	5, 9 or 15	PCL	Casting/Films	DLS, BET, SEM, POM, mechanical tests, molecular dynamics simulation	[127]
CNC	PEG300, PEG-1000, PEG monooleate, Pluronic VR L44	3	PLA	Casting/Films	Turbidity measurements, AFM, TGA, mechanical tests	[117]
CNC	Poly(vinyl acetate), poly(ethylene glycol)	2.4 or 4.8	PHB, PHBV	Melt blending/Films	FTIR, POM, TEM, SEM, AFM, TGA, DSC, mechanical tests	[120]
CNC	PEG (after oxidation with TEMPO)	1–5	PLA/rGO	Casting/Films	FTIR, XRD, SEM, TEM, DMA, TGA, DSC, WVP, tensile and MTT-tests, antioxidant activity	[128]

Methods: TEM: transmission electron microscopy; SEM: scanning electron microscopy; FE-SEM: field-emission scanning electron microscopy; AFM: atomic force microscopy; FTIR spectroscopy: Fourier transform infrared spectroscopy; XRD: X-ray diffraction; GPC: gel-permeation chromatography; TGA: thermogravimetric analysis; DTG: differential thermogravimetric analysis; DSC: differential scanning calorimetry; DMA: dynamic-mechanical analysis; DMTA: dynamic-mechanical thermal analysis; UVis: ultraviolet–visible spectroscopy (transparency); XPS: X-ray photoelectron spectroscopy; WVP: water-vapor permeability; WAXS/WAXD: wide-angle X-ray scattering/diffraction; MTT-test: (3-[4,5-dimethylthiazol-2-yl]-2,5 diphenyl tetrazolium bromide) test; NMR: nuclear magnetic resonance; SBS: short beam shear; ATR-FTIR: attenuated total reflection FTIR; DLS: dynamic light scattering; BET: Brunauer–Emmett–Teller; POM: polarized optical microscopy. Abbreviations: Pluronic: triblock copolymer of poly(ethylene oxide) and poly(propylene oxide); rGO: reduced graphene oxide; CTAB: cetyltrimethylammonium bromide; PEG: poly(ethylene glycol).

Modification of the cellulose nanomaterials with surfactants leads to diminishing the agglomeration of cellulose nanomaterials, and as result to their better distribution in the matrix of hydrophobic polyesters. The homogenous distribution of the filler in the matrix polymer has been observed by many authors [114,116,127].

The properties of modified cellulose and its composite materials are strongly influenced by the chemical nature of the modifying agent. For example, cellulose fibers modified with lignin had no effect on the degradation temperature, while coating with tannin led to a decrease in the fiber degradation temperature [116]. In the latter case, such behavior can be explained by the lower intrinsic thermal resistance of tannin molecules compared to cellulose and lignin. No effect on degradation temperature was observed either for PLA composites filled with CNC bearing adsorbed CTAB [115]. A discussion of the dependence of the mechanical properties of composites on cellulose modification is presented below (see Section 5.2).

In general, the advantages of adsorption as a modification tool are its simplicity and its ability to vary the modifier over a wide range. In turn, the possibility of a leakage of adsorbed molecules from the surface when dispersing the modified cellulose in nonpolar solvents to prepare composites by solution casting or precipitation is a main disadvantage of this approach. As a result, leakage of the modifier from the cellulose surface may affect the properties of the prepared composites, for example, not improving the mechanical properties.

### 4.2. Covalent Modification with Small Molecules

**Figure 5:**
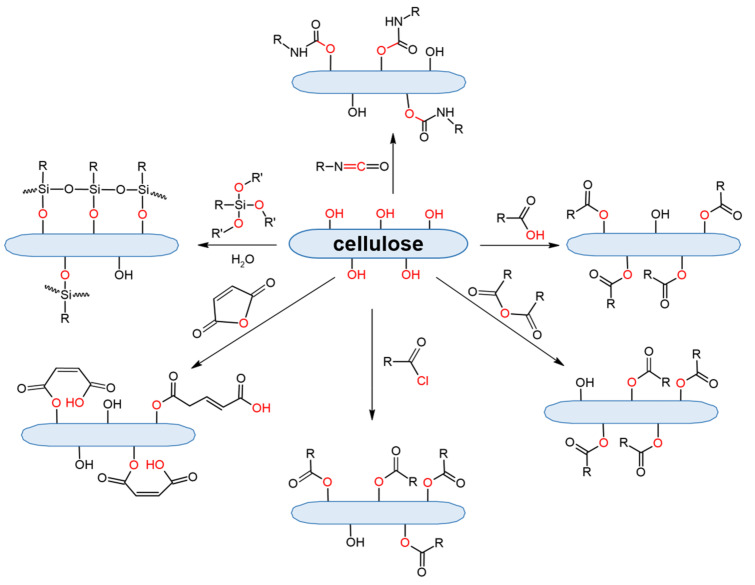
Scheme of cellulose-modification pathways with small molecules (esterification, acylation, silanization, modification with isocyanates).

Cellulose covalent modification is limited by the reactions of its functional groups, namely hydroxyls. In particular, depending on modifier agents, ester, urethane, or silyl ether bonds can be formed due to chemical reactions of cellulose hydroxyls (Figure 5).

#### 4.2.1. Ester Bond Formation

Ester bonds are formed as result of the esterification, transesterification, or acylation of hydroxyls by anhydrides of carbonic acids or acyl chlorides.

a. Esterification and Transesterification

Esterification and transesterification are very simple ways to modify cellulose. Esterification is based on the reaction between hydroxyl and carboxyl groups under strong-acid catalysis (Fischer esterification) to produce ester plus water. The main drawback of this reaction is its reversible nature. In general, to shift the balance toward the direct reaction, the hydroxyls must be in excess relative to the carboxylic acid. The sulfuric acid used in the reaction not only acts as a catalyst, but also serves as a dehydrating agent that binds the released water. Besides sulfuric acid, the esterification can be catalyzed by 4-dimethylaminopyridine (DMAP) as an acyl-transfer catalyst (Steglich esterification).

In the case of cellulose micro- and nanomaterials, the esterification is the second most popular method after adsorption [129,130,131,132,133]. Some of the selected studies are summarized in Table 4. Such carboxylic acids as acetic, butanoic, valeric, dodecanoic, oleic, methacrylic, benzoic, etc., as well as fatty acids have been utilized to hydrophobize cellulose for its further use as a filler for aliphatic polyester matrices. To increase the efficiency of heterogeneous esterification, the modified method involves the activation of cellulose-surface hydroxyls with thionyl chlorides (in organic media) followed by the displacement of thionyl with an appropriate carboxylic acid in the presence of pyridine as a catalyst [134,135,136].

The main methods used for qualitative confirmation are FTIR, and less frequently NMR or XPS (see Table 3). Despite the popularity of this modification approach, only a few studies provided quantitative data on the effectiveness of cellulose modification. Long et al. reported the esterification of cellulose by 70–90% formic acid solution at 70–90 °C for 1–5 h [137]. According to HPLC analysis, the variation of conditions resulted in the binding from 1.7 to 15.8% formyl groups. The maximum amount of formyl groups was introduced during esterification of cellulose at 90 °C for 5 h. At this content of formyl groups, the crystallinity index of modified cellulose fibers was close to that of unmodified fibers. Shojaeiarani et al. modified CNC with valeric acid in DMF in presence of DMAP at 25 °C for 4 h [138]. The reported substitution degree under these conditions was 10% (determined by elemental analysis).

**Table 4:** Selected studies on covalent modification of cellulose micro- and nanomaterials via esterification and transesterification for the preparation of composites with aliphatic polyesters.

Type of Cellulose	Modifying Agent(s)	Filler Content, (wt%)	Matrix Aliphatic Polyester	Processing/ Design of Composites	Characterization Methods	Refs.
CNF and CNC	Acetic acid	1	PLA	Casting/Films	Crystallinity, optical, barrier and mechanical properties	[139]
CNC	Acetic acid	3	PLA	Casting/Films	FTIR, XPS, rheological and mechanical tests, TEM, AFM	[140]
CNC	Acetic acid	3	PCL	Casting/Films	Crystallinity, morphology and mechanical properties	[141]
CMC	Acetic acid	0.25–0.75	PHB	Casting/Films	TD-NMR, XRD, WAXD, TGA, DSC, molecular dynamics	[142]
CMF	Butanoic acid	30	PCL/PCL-g-MAGMA	Melt blending/ Films	FTIR, SEM, XRD, DSC, TGA, mechanical tests	[135]
CNC	Valeric acid	1 or 3	PLA	Extrusion + Molding/ Films	FTIR, TEM, SEM, TGA, DMA, mechanical tests	[138]
CNF	Dodecanoic acid	0.05–1.3	PLA (+PEG as plasticizer)	Melt Spinning/ Fibers	Optical microscopy, SEM, TEM, DSC, mechanical tests	[143]
CNF	Oleic Acid	4, 8 or 12	PLA	Casting/Films	FTIR, SEM, XRD, TGA, DSC, WVP and mechanical tests	[136]
CMC	Methacrylic acid	3 or 10	PLA	Extrusion + Molding/ Films	FTIR, SEM, TGA, DSC, flame retardant and mechanical tests	[144]
CNF	Resin acids (from rosin)	2–10	PLA/ Chitosan	Casting/Films	Elemental analysis, TEM, SEM, mechanical and antimicrobial tests, XPS	[145]
CMC	Palmitic acid (from olive oil)	0.1–2	PLA	Casting/Films	FTIR, XRD, WVP, mechanical, UV and biodegradation tests, TGA	[146]
CNC	Benzoic acid	15	PLA	Casting/Films	TEM, SEM, TGA, DMA and tensile tests	[147]
CMF	Formic acid	1	PLA	Casting/Films	FTIR, SEM, XRD, WVP, moisture adsorption, light transmittance and tensile tests	[137]
CNC	Hexanoic ordodecanoic acid	2 or 7	PLLA, PDLLA	Extrusion and melt spinning/Fibers	SEM, DSC, mechanical tests	[134]
Cellulose fibers	Vinyl laurate	5–30	PLA	Melt blending/ Films	ATR-IR, XPS, XRD, DMA, SEM, DSC, TGA, wettability, rheological and tensile tests	[148]
CNF	Triglycerides of Canola oil	1, 3 or 5	PLA + PBS	Extrusion and Molding/Dumbbells	FTIR, SEM, DSC, TGA, tensile and flexural tests	[42]

Methods: TD-NMR: time-domain nucleic magnetic resonance; UV: ultraviolet spectroscopy (transparency); ATR-IR: attenuated total reflection infrared spectroscopy; for other abbreviations see footnote to Table 3.

Transesterification is based on the displacement of an alcohol from an ester by another hydroxyl-containing compound under acid or basic catalysis. In comparison with esterification this approach is less-used for cellulose modification. The modification of cellulose (nano)fibers with lauryl [148] and fatty-acid residues using this method have been recently reported [42]. The modification of cellulose fiber with lauryl moieties was testified by ATR-IR and XPS.

**Figure 6:**
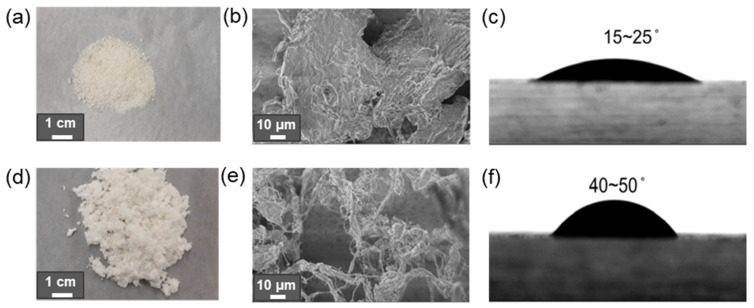
Images of the neat CMF (**a**) and lauryl-CMF (**d**), SEM images of the neat CMF (**b**) and lauryl-CMF (**e**) and contact angles of the neat CMF (**c**) and lauryl-CMF (**f**). Reproduced from [148] with permission of Elsevier.

As one could expect, modification of CNC changes the properties of its dispersion and the final composite materials. Modification of cellulose with small hydrophobic molecules was found to improve the rheological properties by suppressing hydrogen bonds abundant in pure cellulose dispersion [148]. As an example, Figure 6 shows images of contact angle changes and SEM images of before and after CMF modification using vinyl laurate. 

In total, esterification with carboxylic acids is a quite simple process that does not require the utilization of expensive reagents. However, the reversible nature of the reaction demands a thorough selection of reaction conditions to achieve the sufficient degree of modification.

b. Acylation with Anhydrides of Carboxylic Acids and Acyl Chlorides

Another way to form an ester bond between cellulose and the modifying agent is to use anhydrides of carboxylic acids or acyl chlorides. Both are highly reactive toward nucleophiles and can acylate a number of functional groups of biomacromolecules (Figure 5). In these cases, the carboxyl is activated and the acylation of hydroxylic groups is facilitated compared to the use of carboxylic acids.

If the anhydride is formed from monocarboxylic acids (e.g., acetic anhydride), acylation requires a deprotonating agent (e.g., pyridine) and is accompanied by the release of one acid molecule as a byproduct. In turn, reactions with dicarboxylic acid anhydrides (e.g., succinic or maleic anhydride) undergo nucleophilic ring opening to form an acylated product containing a newly formed carboxylate group under elevated temperatures. In turn, acyl chlorides have a greater reactivity in comparison with anhydrides. Despite the high reactivity of acyl chlorides compared to other carboxylic acid derivatives (anhydrides, esters, amides), hydroxyl acylation using them can be further promoted by the addition of organic bases such as pyridine. The latter acts as a catalyst by forming an active intermediate with the carbonyl group of acyl chloride. The summary of the application of anhydrides of carboxylic acids and acyl chlorides for cellulose modification is presented in Table 5.

**Table 5:** Summary on covalent modification of cellulose micro- and nanomaterials via acylation with anhydrides of carboxylic acids and acyl chlorides for the preparation of composites with aliphatic polyesters.

Type of Cellulose	Modifying Agent(s)	Filler Content (wt%)	Matrix Aliphatic Polyester	Processing/ Design of Composites	Characterization Methods	Refs.
CNF	Acetic anhydride	1 or 2	PCL/ Gelatin	Electrospinning/ Nanofibrous scaffolds	FTIR, SEM, WAXS, DSC, biodegradation, conductivity and mechanical tests	[149]
CNF	Acetic anhydride	5	PLA	Extrusion/Strands	FTIR, XRD, TGA, SEM, DMA, tensile and wettability tests	[150]
CNF	Acetic anhydride	0.2–3	PLA	Casting/Films	FTIR, UV, SEM, DSC, mechanical tests	[151]
CNF	Acetic, propionic or butyric anhydride	2	PLA	Casting/Films	ATR-IR, SEM, DSC, wettability, transmittance, transparency, and mechanical tests	[152]
CMF	Acetic anhydride	1–20	PLA/ PLA-EGMA	Casting/Films	FTIR, XRD, TGA, optical microscopy, wettability and mechanical tests	[153]
Cellulose fibers	Acetic anhydride	20, 30 or 40	PLA	Extrusion + molding/ Films	FTIR, TGA, kinetics study	[154]
CNC	Succinic anhydride	1, 2 or 3	PLA	Extrusion + molding/Films	FTIR, TEM, SEM, DSC and DMA	[155]
CNF	Maleic anhydride	5–10	PLA	Casting/Films	FTIR, XRD, SEM, TEM, TGA, mechanical tests	[156]
CNC	Maleic anhydride	1, 3 or 5	PLA	Casting/Films	FTIR, XPS, FE-SEM, DMA and tensile tests	[157]
CNC	Maleic anhydride and furan methylamine	1	PCL/TPU	Extrusion/Filaments	FTIR, shape memory, self-healing, conductivity study, molecular dynamics simulations	[158]
Mixture of celluloses and lignin fibers	Maleic anhydride or APTES	5	PLA	Extrusion + molding/ Films	FTIR, SEM, EDX, wettability and mechanical tests	[159,160]
CMC	Butyryl or lauroyl chlorides	0–9	PLA/BS CMC	Extrusion/ Pellets and Films	NMR, FTIR, SEM, TGA, and mechanical tests	[161]
CNF	Stearoyl chloride	30	PLA	Melt blending/ Blends	DSC, hardness, rheological, wettability and mechanical tests	[162]
CNC	Dodecanoyl chloride or APTES	0.5, 1 or 2	PLA	Extrusion + molding/ Films	ATR-IR, AFM, SEM, XRD, wettability and mechanical tests	[163]
CNC	Palmitoyl chloride	0.5 or 1	PHBV	Melt blending	SEM, HSPOM, TGA, DSC, rheological and mechanical tests	[164]
Lignincellulose	Benzoyl chloride	1–5	PLA	Casting/Films	FTIR, DSC, XRD, SEM, DMTA, rheological and tensile tests	[165]

Methods: HSPOM: hot-stage polarized optical microscope WAXS: wide-angle X-ray scattering; EDX: energy-dispersive X-ray spectroscopy. Abbreviations: APTES: (3-aminopropyl)triethoxysilane; EGMA: ethylene–glycidyl methacrylate copolymer; TPU: thermoplastic polyurethane; BS CMC: banana leaf sheath cellulose microcrystals; for other abbreviations see footnote to Table 3 and Table 4.

Under proper conditions, these reactions provide good modification efficiency. For instance, Jamaluddin et al. studied a modification of CNF (in DMF) with acetic, propionic, and butyric anhydrides within 4 h to investigate the reaction rate [152]. Using EDX analysis, the degree of substitution with acetic and propionic anhydride was found to be quite close (around 30%), while the butyric anhydride demonstrated lower modification efficacy (~24%). A 20% acetylation degree of CNF (determined by FTIR) when modified with acetic anhydride in DMF in the presence of pyridine at 105 °C for 20 h was found by Zepic et al. [151]. The average diameter of pure CNF was 43 nm, whereas for acetylated CNF it was 55 nm. In addition, pure CNF was found not to disperse in chloroform, forming large aggregates due to hydrogen bonding. At the same time, acetylated CNF effectively dispersed after 2 min of sonication. 

Sojoudiasli et al. reported the modification of CNC with myristoyl chloride in 1,4-dioxane in presence of 1-methylimidazole as a catalyst during 7 h [166]. By applying such conditions, the authors managed to achieve a 31% hydrophobic carboxylic acid coverage of the CNC surface. At the same time, Zhou et al. prepared the cellulose nanocrystals modified with maleic anhydride for reinforcement of the PCL-based composites. Using XPS analysis, the authors detected only about 10% carboxylate groups in the modified CNC samples after the modification, which proceeded in DMF at 120 °C for 20 h under stirring [157].

**Figure 7:**
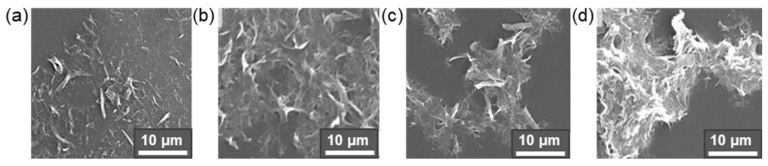
SEM images of CNF before (**a**) and after modification with acetic (**b**), propionic (**c**), and butyric (**d**) anhydrides (reaction time was 4 h). Reproduced from Supplementary Materials of [152], published under the terms of the Creative Commons CC BY license.

As several studies have shown, the modification of the cellulose surface with small hydrophobic molecules affects also the crystallinity of micro- and nanomaterials. For instance, Szefer et al. studied the effect of CNC modification with succinic anhydride on the crystallinity fraction content [155]. It was found that the modified CNC showed a higher content of the crystallinity fraction (79.4%) compared to the neat CNC (74.9%). The authors supposed that the increase in crystallinity could be advantageous for the reinforcement of PLA using modified CNC as a filler. Jamaluddin et al. determined the degree of crystallinity (by DSC method) for pure PLA and its composites with neat and hydrophobized CNF. For all composites, the degree of crystallinity was lower than that of pure PLA (39.6%) [152]. However, while this parameter was in the range of 28.2–33.1% for composites with modified CNC, it was only 16.2% for the composite with pure CNC. The reason for such a result was the incompatibility between the neat CNF filler and the PLA matrix, that, in turn, promotes the formation of more aggregates than with the modified CNF. At the same time, the length of the modifying agent, namely acetic, propionic, or butyric anhydride, had no significant effect on the degree of crystallinity. The effect of using anhydrides with different chain lengths on CNF morphology is illustrated in Figure 7.

Like cellulose materials prepared by chemical modification by (trans)esterification, cellulose hydrophobized by acylation with anhydrides and acyl chlorides also shows good compatibility with hydrophobic matrices. Studying the ultrathin sections of composite materials by TEM, Fumagalli et al. indicated the efficient dispersion of CNC and CMF hydrophobized with the use of myristoyl chloride in comparison with neat cellulose materials [167]. In particular, efficient dispersion of modified CNC in a hydrophobic matrix was achieved with a volume fraction of dry nanocellulose filler up to 20 wt%. A similar result was also observed by Bin et al. [153]. While neat CMF was only able to be introduced into PLA in an amount of 3 wt%, in the case of acetylated CMF, the amount of filler was increased up to 20 wt%. The improved interfacial compatibility has a positive effect on the mechanical properties of the composites (see Section 5.2).

In comparison to esterification with carboxylic acids, acylation with anhydrides of carboxylic acids and acyl chlorides can provide higher degrees of surface modification. In general, ester bonds formed between the modifier and cellulose are suitable for the preparing composites with aliphatic polyesters using any processing technique. At the same time, the sensitivity of the ester bonds to hydrolysis can play a positive role in the degradation of composite materials, e.g., in the case of packaging ones.

#### 4.2.2. Silyl Ethers Formation

Silyl ethers represent chemical bonds consisting of Si covalently bonded to an alkoxy group (-C-O-Si-) due to the reaction between organosilanes and hydroxyl groups (Figure 5). Silanization is an inexpensive and effective method for modifying organic and inorganic surfaces enriched with hydroxyls. Unlike previous modifications, silanization of cellulose materials is used both as an independent functionalization of cellulose and as an intermediate one to introduce the functionality needed for further modification. A wide variety of different silane agents are now available, representing mainly functional ethyl/propyl tri(methoxy/ethoxy)silane agents [168]. Generally, alkoxysilanes have a low reactivity to hydroxylic groups under ambient temperature conditions. In comparison, trimethoxysilanes are more reactive than triethoxysilanes. While trimethoxysilanes react slowly with OH-groups at room temperature, triethoxysilanes are practically unreactive without prior hydrolysis. At the same time, under the right conditions both are sufficiently reactive and can modify OH-bearing surfaces without prior hydrolysis. In particular, the addition of a basic or acidic catalyst increases the reaction rate by increasing the hydrogen-bonding capability of surface hydroxyls and facilitates the reaction at room temperatures.

Selected studies on the cellulose modification by silanization are listed in Table 6. As seen, (3-aminopropyl)triethoxysilane (APTES), (3-methacryloyloxypropyl)trimethoxysilane (MPTMS), and (3-glycidoxypropyl)triethoxysilane (GPTES) are among the most-used silane agents. The effect of variation in silane agent amount on the properties of cellulose micro- and nanomaterials and their composites with aliphatic polyesters has been studied by several research groups [169,170,171,172,173].

**Table 6:** Selected studies on covalent modification of cellulose micro- and nanomaterials via silanization for the preparation of composites with aliphatic polyesters.

Type of Cellulose	Modifying Agent(s)	Filler Content (wt%)	Matrix Aliphatic Polyester	Processing/ Design of Composites	Characterization Methods	Refs.
CMC	APTES	3	PLA/PP	Extrusion + Molding/Films	FTIR, SEM, DSC, rheological, DMA and tensile tests	[174]
CMC	APTES	0–25	PLA	Automated coating/Films	FTIR, XRD, SEM, TGA, degradation study and mechanical tests	[171]
CNF	APTES	9.5 or 17	PCL	Electro-spinning and compression molding/Films	SEM, XPS, DSC, DMA and tensile tests	[175]
CNC	APTES	2.5	PLA	Compression molding/Sheets	FTIR, SEM, DMTA	[176]
Cellulose fibers	APTES	6, 8 or 10	PLA-co-glycerol	Impregnation of filler into resin/Slides	TGA, DSC, SEM, flexural, wettability, water adsorption, conductivity, DMTA and mechanical tests, element and resonance analysis	[169]
Cellulose fibers	APTES	30	PLA	Blending	FTIR, SEM, DSC, HDT, mechanical tests	[172]
CNC	CETMS	0.5 or 1	PLA	Hot pressing/Films	FTIR, FE-SEM, WAXS, mechanical tests	[177]
CNC	MTMS	2.5	PLA	Casting/Films	FTIR, SEM-EDS, TEM, TGA, DSC, mechanical tests	[178]
CMF	APTES, DMS and TMS	1	PLA	Extrusion + molding/blends	NMR, FTIR, DSC, TGA, and mechanical tests	[179]
CNF	MPTMS	0.25–2	PLA	Casting; Melt blending/Films	NMR, FTIR, SEM, AFM, TGA, and mechanical tests; XPS, DSC	[170]
CNW	MPTMS, APTES, VTMS, MTMS	2.5; 3	PLA	Casting/Films	FTIR, DSC, SEM, and mechanical tests	[173,180]
CNC/DPF	MPTMS and PEG-6000	N/A	PLA	Hot molding/Strips	FTIR, SEM, TGA, DSC, water adsorption, degradation and mechanical tests	[181]
CNF	VTMS, APTES and GPTES	5	PLA	Extrusion + Molding/Films	FTIR, TGA, AFM, SEM, and mechanical tests	[182]
Cellulose fibers	GPTES	30	PLA + PP	Hot molding/Films	FTIR, SEM, XRD, TGA, DMA	[183]
Lignincellulose fibers	MPTMS vs. acetic anhydride	30	PLA	Extrusion/Strands	FTIR, TGA, SEM, GPC, TGA, mechanical tests	[184]

Methods: HDT: heat deflection temperature; for other abbreviations see footnote to Table 3. Abbreviations: DPF: digital printing wastepaper fiber; APTES: γ-aminopropyltriethoxysilane; CETMS: 2-(carbomethoxy)ethyltrimethoxysilane; MTMS: (3-mercaptopropyl) trimethoxysilane (A-189); DMS: N-(2-aminoethyl)-3-aminopropyltrimethoxysilane; TMS: N^1^-(3-trimethoxysilyl propyl) diethylenetriamine; MPTMS: 3-methacryloxypropyltrimethoxysilane; VTMS: vinyltrimethoxysilane; VTES: vinyltriethoxysilane; GPTES: glycidyloxypropyl triethoxysilane.

**Figure 8:**
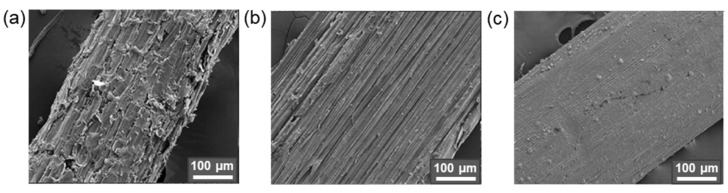
SEM images of untreated cellulose fibers (**a**), treated by 10 wt% NaOH solution (**b**) and treated by 10 wt% NaOH solution and 2 wt% of APTES (**c**) (reproduced from [172] with permission of John Wiley & Sons).

For example, Qu et al. modified CNF with MPTMS at room temperature within 1 h. The determined degrees of substitution were 2.05, 3.84, 5.90, and 6.84% for initial amounts of MPTMS equal to 0.5, 1.0, 1.5, and 2.0 vol% relative to 1 wt% CNF suspension in ethanol. The best mechanical properties were detected for composite prepared by the casting of the PLA solution containing CNF modified by MPTMS (1 vol%): the highest tensile strength and elongation at break increased by 42.3% and 28.2%, respectively, compared to pure PLA. In turn, Li at al. reported the modification of CMC with APTES in 90% ethanol in the presence of a catalytic amount of glacial acetic acid (pH 4–5) at room temperature [171]. The amount of APTES varied from 0.5 to 4.4 wt% relative to CMC. As in the previous case, the tensile properties depended on the amount of silane agent used for modification. The maximum values of tensile strength and elongation at break were found for the PLA composites reinforced with CMC modified with 3 wt% APTES. At the same time, according to XRD analysis, the crystallinity index was decreased during modification with APTES by approximately 5% in comparison to neat CMC. Figure 8 demonstrates images of cellulose fiber, clearly indicating the change in surface morphology after alkaline etching and modification with APTES [172].

Araujo et al. used three different silane agents, namely APTES, GPTES, and VTMS to modify CNF [182]. The modification was carried out in a mixture of ethanol–water (90:10 v/v) at 60 °C for 24 h. The silanization of CNF with organosilanes was proven using NMR spectroscopy. However, quantitative data on the effectiveness of modification with this or that silane were not presented. A comparison of the mechanical properties of PLA filled with CNF modified by different silanes showed a slight increase in Young’s modulus for VTMS and GPTES modification and a more evident increase in stress at break for APTES modification. An analogous study, but also with the use of MTMS for modification of cellulose nanowhiskers, was performed by Ma et al. [173]. Silanization was fulfilled in a water–alcohol solution (water/alcohol = 80/20) under acidic catalysis (pH 4 adjusted with acetic acid) at concentrations of silane agents equal to 1, 8, and 16 wt% and 50 °C for 30 min. Regardless of the silane agent, the best tensile properties were established for PLA films produced by solution casting and filled with CNW modified with 8 wt% silane. The elongation at break increased significantly from 12.4% for untreated CNW to approximately 214, 255, and 210% for VTMS, MPTMS, and MTMS treatments, respectively, while APTES demonstrated a relatively weak improvement in strength (111 %). A similar effect was recently observed for composites produced by compression molding and based on PCL containing CNF modified by APTES: cellulose modification had virtually no effect on tensile strength and elongation at break, and only a slight improvement was found for Young’s modulus [175]. Virtually no improvement in mechanical properties was observed in the work of Zhang et al. who prepared composites by hot molding from PLA, digital printing waste paper fibers (DPF), and CNC modified with APTES [181]. Most likely, such a dramatic difference in the properties of composites fabricated with the use of silanized cellulose micro- and nanomaterials is strongly related not only to the properties of different silanes, but also to the effectiveness of the cellulose modification, which, unfortunately, is not given sufficient attention in the recent studies.

#### 4.2.3. Urethane-Bond Formation

Urethane or carbamate bonds are formed by the reaction of isocyanate groups with compounds containing active hydrogen atoms, such as in hydroxyl groups (Figure 5). Isocyanates are highly reactive compounds, showing the highest reaction rate at alkaline pH values (e.g., pH 8.5). The main drawback of isocyanates is their high sensitivity to hydrolysis, since moisture rapidly decomposes them. Thus, many reaction protocols recommend performing the reaction with isocyanates in an organic medium (e.g., DMSO, toluene, etc.) [168].

Modification of cellulose with isocyanates is not as popular as acylation for forming an ester bond, and nor is silanization. Nevertheless, there are several recent papers describing such modification for preparation of the aliphatic ester/cellulose composites (Table 7). Among the isocyanates used are aliphatic mono- and bifunctional compounds such as *n*-octadecyl isocyanate and isophorone diisocyanate, and aromatic compounds such as toluene-2,4-diisocyanate.

**Figure 9:**
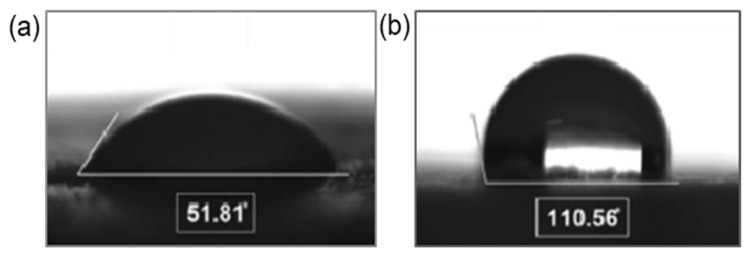
Contact angles of neat CNC (**a**) and CNC modified with isophorone diisocyanate (**b**) (reproduced from [188] with permission of Elsevier).

The modification of cellulose micro- and nanomaterials with *n*-octadecyl isocyanate is reported to be performed in toluene at 90–110 °C for 30 min [29,185]. Recently, Ogunsona et al. studied the CNC modification with isophorone diisocyanate in toluene, toluene/DMSO mixture and DMSO at 105 °C for 1 h [188]. According to the elemental analysis, a higher coupling efficiency was achieved using toluene/DMSO = 10/90 (11%) or pure DMSO (10%). Figure 9 demonstrates the change in the CNC wettability as a result of the surface hydrophobization [188].

**Figure 10:**
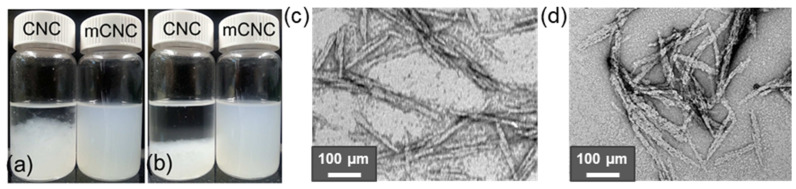
Images of neat CNC and modified CNC (mCNC) in chloroform after 15 min sonication (**a**) and after 15 min sonication and standing (**b**); TEM images of neat (**c**) and modified (**d**) CNC (reproduced from [186] with permission of Royal Society of Chemistry).

Olonisakin et al. investigated the substitution degree in the CMC modification reaction with toluene-2,4-diisocyante [32]. The reaction was performed in THF at 75 °C for various times (from 1 to 24 h). The determined substitution degree ranged from 11 to 16% and reached 14% after 10 h. A change in the hydrophilic–hydrophobic properties of CNC after modification with toluene-2,4-diisocyanate [186] is shown in Figure 10.

In sum, the reactivity of isocyanates is excellent and the reaction with hydroxyls run quite fast. Compared to other modifications, the carbamates, formed by modifying cellulose with isocyanate-bearing molecules, are extremely stable bonds. Thus, the probability of surface-modifier leakage during further manipulations with the modified filler is minimized.

#### 4.2.4. Other Modifications

Some other ways to modify the surface of cellulose micro- and nanomaterials with low-molecular compounds [190,191,192,193,194,195] are also reported. For instance, the activation of carboxyl groups (introduced by TEMPO-mediated oxidation of cellulose hydroxyls) allowed the further attachment of octadecylamine to form an amide bond between cellulose and a modifier [191]. The synthesis of hexyl-CNF can be carried out by two-step method, which includes the reaction of CNF hydroxyls with mono-chloroacetic acid to form carboxymethylated CNF followed by substitution of carboxymethyl-moiety with hexyl one under acidic catalysis as described by Eyholzer et al. [193]. Given the high reactivity of hexahydroxyl N-containing heterocyclic compounds, Yin et al. used 2,4,6-trichloro-1,3,5-triazine as a crosslinking agent for grafting dodecylamine onto the surface of CNC [194].

In order to provide conductive properties to cellulose-based materials, the surface of cellulose can be modified by metals [196,197,198]. In particular, Sundar et al. reported the modification of CMC with Fe (II) ions by the reaction of iron hydroxide obtained in situ with carboxylic groups generated in CMC via oxidation of surface hydroxyls [196]. Another approach to introduce Fe (II)-ion cellulose materials was proposed by Hassan et al. [197]. They used 4′-chloro-2.2′:6,2”-terpyridine to introduce the terpyridine-chelating group into the surface of CNC. Introduced terpyridine groups are capable of binding Fe (II) ions during further treatment with FeSO_4_. To prepare the electroactive material, Ummartyotin et al. developed a protocol for the functionalization of bacterial cellulose with Sr ions [198]. For this, the cellulose suspension was treated with strontium chloride at an elevated temperature for 3 h to react with cellulose hydroxyls and finally to form Sr-O bonds.

### 4.3. Covalent Modification with Polymers

Recently, the functionalization of cellulose with polymers has become a new tool for modifying the properties of cellulose as a filler for aliphatic polyesters in order to improve the mechanical, thermal, or biological properties of composites. There are two techniques that can be used for such modification, namely polymer grafting from the surface of cellulose micro- or nanomaterials by in situ polymerization (the grafting “from” method), or covalent immobilization of presynthesized polymers on the surface of cellulose materials (the grafting “to” method). Summarized results on cellulose modification using both grafting “from” and grafting “to” approaches are discussed below.

#### 4.3.1. Grafting “from”

**Figure 11:**
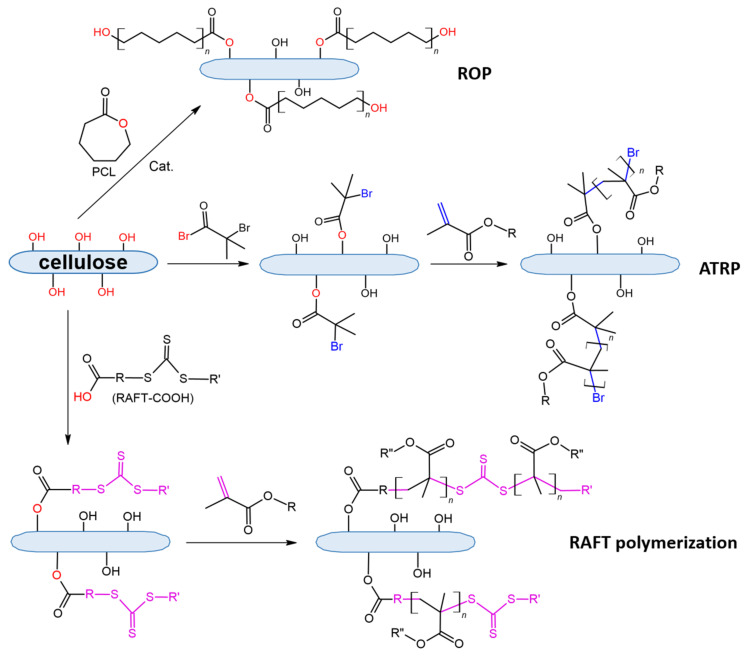
Examples of cellulose modification by the grafting “from” technique (the details and corresponding references are presented in Table 8).

The presence of a large number of hydroxyl groups on the surface of cellulose materials makes it extremely attractive for surface-initiated ring-opening polymerization for cyclic monomers of aliphatic hydroxy acids, e.g., lactide, glycolide, and lactones. Indeed, the first works devoted to the grafting of polymers from the cellulose materials focused on the ROP of ε-caprolactone [67,199] and L-lactide [199] initiated by surface hydroxyls (Figure 11). The application of surface-initiated ROP makes it possible to obtain cellulose grafted with aliphatic polyesters, which are ideal candidates for increasing the compatibility of the filler with the aliphatic polyester matrix. Thus, it is not surprising that by now the modification of cellulose micro- and nanomaterials of PLA and PCL, followed by the production of composites and the evaluation of their properties, has been studied and discussed by many authors (Table 8).

The main feature of ROP in the grafting from the cellulose surface is the heterogeneous nature of this process. A typical procedure of such grafting involves the utilization of stannous (II) octoate as a catalyst (0.2–2.0 wt% with respect to the monomer) and performing the reaction in toluene at 80–130 °C for 18–24 h [67,199,202,205,208]. Chai et al. reported the PLLA and PDLA synthesis in toluene at 170 °C for 8 h [201]. In all cases, the success of the grafting was confirmed by FTIR and in some studies by solid-state NMR and XPS (Table 7). Measurement of the contact angles for the cellulose substrate grafted with PLLA and PCL showed the increase in this parameter after modification. The grafting of short chains of PCL and PLLA resulted in a contact angle equal to 95 and 107 °, while the long chains of the same polymers provided contact angles of 99 and 112 °, respectively [199].

Recently, Chuensangjun et al. reported a chemo-enzymatic preparation of CNC grafted with PLA [219]. The developed procedure included (1) the oxidation of CNC surface with TEMPO, (2) ROP of L-lactide catalyzed with stannous (II) octoate, and finally (3) additional esterification of the CNC surface hydroxyls with the oligomer of lactic acid obtained by lipase-catalyzed ROP. Depending on the reaction conditions (temperature and reaction time), the percentage of grafting ranged from 3.4 to 59.6%. Optimal conditions for ROP included a two-temperature protocol: 15 min at 140 °C and 8 h at 100 °C, followed by incubation of CNC-g-PLA with the enzymatically produced oligomer of lactic acid for an additional 16 h at 100 °C. Under these conditions, the percentage of grafting was maximal along with the high crystallinity of CNC-g-PLA sample (>76%).

The use of stannous (II) octoate as a catalyst does not satisfy the requirements of green chemistry and may remain in the polymer, requiring further purification steps for possible sensitive applications, such as biomedical applications. In this regard, the development of novel grafting methods that exclude the use of stannous (II) octoate is in high demand. One such method was recently proposed by Yoo et al. who used zinc acetate dihydrate to catalyze the surface-initiated polycondensation of D,L-lactic acid taken as aqueous syrup (85 wt%) containing CNC (5 wt%) at 180 °C [220]. In addition, the authors replaced some of the lactic acid with dodecanoic, palmitic, or stearic acids for common polycondensation in the presence of dibutyl tin dilaurate catalyst at 190 °C and 100 mmHg for 30 min, and then at 35 °C and 10 mmHg until the viscous solution was obtained. Using NMR spectroscopy, the degree of polymerization was calculated. The average DP for PLA grafting was 6, while for copolycondensation with palmitic acid it reached 8.

Labet et al. offered the use of citric acid as an alternative to metal catalysis in the production of cellulose grafted with PCL [212]. The reaction conditions were optimized with respect to ratio between ε-caprolactone, citric acid, and cellulose-surface hydroxyls as well as temperature and polymerization time. According to the XPS analysis, the most successful grafting occurred under the following conditions: [ε-caprolactone]:[citric acid]:[surface OH] = 660:10:1, 150 °C and 2 h. Under optimized conditions, the amount of grafted PCL corresponded to 58 wt%.

In addition to PLA and PCL, the successful grafting of PBS from CNC via polycondensation of 1,4-butanediol and succinic acid with cellulose surface hydroxyls was recently reported by Zhang et al. [214]. The reaction was carried out at 220 °C for 4 h at normal pressure in the presence of titanium butoxide (0.1 wt% of the reactants). SEC analysis of PBS dissociated from CNC allowed the determination of the PBS molecular weight. The highest determined *M_n_* was 23,700.

**Figure 12:**
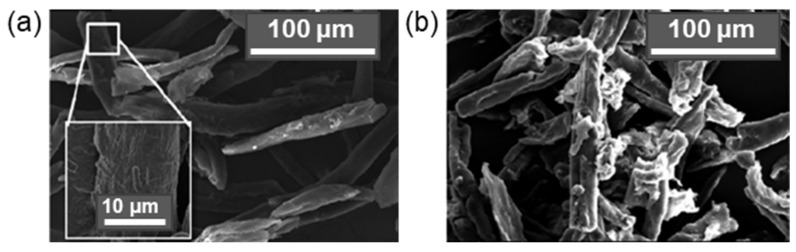
SEM images of CMC before (**a**) and after grafting of oligo(L-lactic acid) (**b**). Reproduced from [82] with permission of Royal Society of Chemistry.

Previously considered studies included the direct grafting of aliphatic polyesters from the surface hydroxyls of cellulose materials. To increase the amount of bound polyester, Peng et al. premodified cellulose with APTES and then with 3,5-diaminobenzoic acid [213]. This approach made it possible to obtain a corona with multiplied amino groups. Thereafter, ROP of D,L-lactide was carried in DMSO in the presence of stannous caprylate as a catalyst at 130 °C for 16 h. As a result, a hyperbranched corona of PLA was produced on the surface of CNC, as speculated by the authors. CNC modification with PLA was confirmed by FTIR spectroscopy. However, the authors did not provide any quantitative data to support the idea that this approach was superior in comparison to PLA grafting initiated by CNC surface hydroxyls. As an example, a change in the morphology of the CMC surface due to the grafting L-lactide acid oligomers is illustrated in Figure 12 [82].

Besides aliphatic polyesters, several research groups used radical polymerization as a tool to functionalize the cellulose surface with various poly(meth)acrylates. The realization of such an approach required the cellulose with a small molecule capable of further polymerization. Following this idea, the adsorption of monomers on the cellulose surface, its silanization with vinyl-, methacryloyl-, or glycidoxy-containing silanes, or treatment with α-bromoisobutyryl bromide were implemented. The adsorption of butyl acrylate (BA), 2-ethylhydroxy acrylate (HEA), and methylmethacrylate (MMA) was the oldest approach proposed for modifying the cellulose fibers with poly(meth)acrylates [215]. Despite the simplicity of this approach, possible polymer leakage from the fiber surface limits the application of such a technique. However, recently, a similar approach was used to modify agave cellulose fibers covalently with PMMA [221]. A decrease in crystallinity from 80% to 68% was reported after PMMA grafting with an efficiency of 55%.

Popa et al. described the use of two silane agents containing vinyl (VTES) or methacryloyl (MPTMS) moieties, for modification of CMF for further radical polymerization of MMA [218]. In both cases, the PMMA grafting was evidenced by FTIR. At the same time, no direct data on the grafting efficiency was reported. Both samples blended with PHB had very similar thermal properties but different tensile strengths. Composites of PHB with CMF grafted through silanization with MPTMS showed 30% better tensile strength, 60% better elongation at break, and 15% better Young’s modulus than composites with CMF pretreated with VTES. This fact indirectly indicates a better grafting of PMMA when MPTMS is used to premodify cellulose. The results seem to be expected, since MMA, being a methacrylate type monomer, polymerizes better with methacrylate-type silane because of the similar double-bond activity in these monomers.

An original approach to grafting a methacrylate-type polymer from the CNC surface by controlled radical polymerization was proposed by Boujemaoui et al. [216]. A procedure for pretreatment of CNC with α-bromisobutyryl bromide to obtain CNC-Br was developed. The latter was used for the surface-initiated ATRP of BMA (Figure 11). The amount of PBMA grafted from CNC was found to be 4 or 28% for low (degree of polymerization 110) and high (degree of polymerization 487) molecular weights, respectively.

Another example of the controlled radical polymerization on the surface of cellulose was reported by Aubin et al. [222,223]. In this case, the functionalization of CNC with poly(N-isopropylacrylamide) (PNIPAM) and N,N’-dimethylaminoethyl methacrylate (DMAEMA) by RAFT polymerization was employed (Figure 11). Before polymerization, the CNC surface was modified with a chain-transfer agent (4-cyano-4-[(dodecylsulfanylthiocarbonyl)sulfanyl]pentanoic acid) (CTA) under carbodiimide activation and in presence of DMAP as a catalyst in DMF at 50 °C for 48 h. Three different grafting densities, namely 0.006, 0.09 and 0.46 CTA/nm^2^, were achieved by varying the initial CTA concentration [222]. The copolymer grafting was carried out in DMF at 70 °C for 72 h using AIBN as an initiator by varying the DMAEMA concentration from 0 to 20 mol%. According to NMR analysis, the molecular weights of the grafted copolymer were close to the theoretical ones, confirming the controlled nature of polymerization. The NIPAM conversion ranged from 80 to 96%, while the degree of polymerization varied from 330 to 1900 depending on the ratio of reactants [222].

Thus, the examples overviewed in this subsection illustrate a diversity of techniques, such as ring-opening polymerization, and free-radical and controlled radical polymerizations, which can be used to modify the surface of cellulose micro- and nanomaterials by a grafting “from” technique. Biodegradable (PLA, PCL, PBS), nondegradable hydrophilic (PHEA) and hydrophobic (PBA, PMMA, PBMA), as well as thermoresponsible (PNIPAM) polymers can be successfully grafted.

#### 4.3.2. Grafting “to”

**Figure 13:**
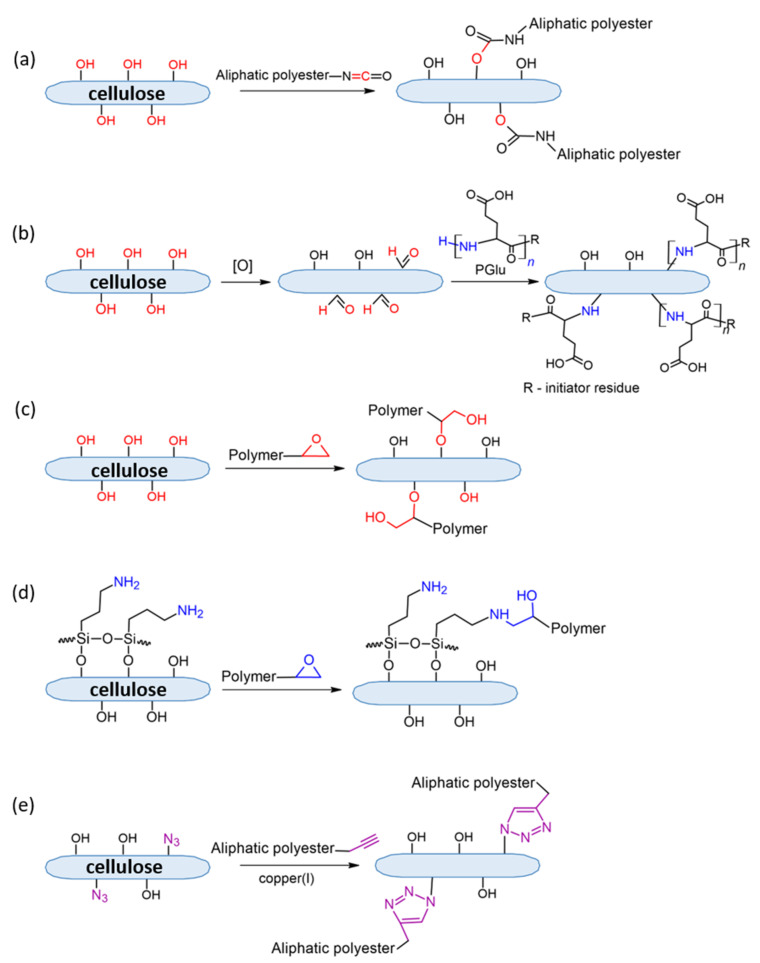
Possible ways of modifying the cellulose surface with macromolecular compounds by grafting “to” (Table 9): (**a**) attachment of polymer containing isocyanate group, (**b**) interaction of polymer amines with aldehyde groups of preoxidized cellulose, (**c**,**d**) modification of cellulose with epoxy-bearing polymers, (**e**) modification via “click”-reaction.

In contrast to the previous approach, grafting “to” is used to modify the surface of cellulose materials with presynthesized polymers. Figure 13 illustrates the reported pathways to modify cellulose materials with polymers via a grafting “to” technique.

One of the first described methods of grafting “to” is the modification of cellulose with PLA via its intermediate functionalization sequentially with phenyl isocyanate and toluene diisicyanate in a mixture of anhydrous methylene chloride and anhydrous toluene under reflux for 4 h. The resulting intermediate was reacted with CMC or bleached kraft softwood pulps during 72 h for modification [224]. Recently, a similar approach was used to modify CNC with PLLA. The reaction also involved two steps, namely activation of the polyester in DMSO at 60 ℃ for 3 h followed by reaction with CNC hydroxyls in DMSO at 120 ℃ for 12 h [207]. In both cases, the modification was evidenced by FTIR and NMR [207] or XPS [224], but neither study contains quantitative data on the effectiveness of grafting.

Besides aliphatic polyesters, poly(amino acids) can be grafted onto the cellulose surface. Recently, Averianov et al. reported on the modification of CNC with hydrophobized poly(glutamic acid) (PGlu) via two-step approach [225]. It was based on (1) the partial oxidation of cellulose vicinal diols to aldehyde groups and (2) their reaction with terminal amino groups of PGlu. The authors compared the grafting of PGlu of two molecular weights (10,400 and 2,100) and found a better modification in the case of PGlu with a lower molecular weight. In this case, 90 wt% of PGlu taken for the reaction was bound to CNC.

Hong et al. reported the direct modification of CMC with poly(ethylene-co-glycidyl methacrylate) (PEGMA) by the reaction of epoxy groups of the polymer with cellulose hydroxyls [226]. The reaction was carried out in xylene at 120 °C for 3 h under acid catalysis with *p*-toluenesulfonic acid. The success of the modification was proven by both XPS and FTIR spectroscopy. Premodification of CNC with APTES allowed further successful grafting of epoxy-bearing PEG. The modification of cellulose hydroxyls with epoxy group of allyl glycidyl ether, the further epoxidation of a double bond, and the attachment of PVA through its hydroxyls was reported by Virtanen et al. [190]. The reaction between CNC-APTES- and epoxy-PEG was run in water at 65 °C for 6.5 h. According to the elemental analysis (XPS), the grafting efficiency of PEG to CNC was calculated to be 43.36% [227].

In addition, click chemistry may be also an option if grafting of presynthesized polymers is of interest. Krouit at al. reported the modification of cellulose fibers with 10-undecynoic acid by esterification and used azide-PCL to perform a Cu(I)-catalyzed heterogenous click reaction in THF at room temperature for 48 h [228]. The azidation was confirmed by FTIR, XPS, and NMR spectroscopy. The yield of the azidation reaction was over 90%. Another means of click-chemistry performance was recently proposed by Mincheva et al. [229]. In this case, the authors used azidized CNC and propyl-bearing PLLA for azide-alkyne cycloaddition in THF at 50 °C under copper(I) catalysis. The amount of PLLA grafted onto the CNC surface was calculated to be 12 wt%.

**Table 9:** Summary on covalent modification of cellulose micro- and nanomaterials by grafting “to” technique.

Type of Cellulose	Grafted Polymer	Cellulose Premodification/Polymerization Technique	Filler Content (wt%)	Matrix Aliphatic Polyester	Processing/ Design	Characterization Methods	Refs.
CNC	PGlu	Amination/ROP	5	PLLA	Casting/Films	NMR, DLS, TGA, mechanical tests	[225]
CNC	PGlu	Amination/ROP	5, 10 or 15	PLLA, PDLLA, PCL	Casting/Films	OTM, ORM, SEM, POM, mechanical tests, MTT-test, in vivo study, histology	[230,231]
CNC	PLA	Toluene diisocyanate	0.2, 0.5 or 1	PLA	Casting/ Sheets	NMR, FTIR, GPC, TEM, SEM, DSC, TGA, rheology study	[207]
CNC	Propargyl-containing PLA/PBS	Tionyl chloride followed with sodium azide	−	−	−	SEC, MALDI, ATR-IR, XPS, NMR, TGA, SEM	[229]
Cellulose fibers	N_3_-PCL	10-undecynoic acid	−	−	−	FTIR, NMR, XPS, elemental analysis	[228]
CNC	Epoxy-PEG	APTES	1–5	PLA	Hot pressing	FTIR, TEM, XPS, XRD, SEM, POM, TGA, DSC, wettability and mechanical tests	[227]

Methods: OTM: optical transmitted microscopy; ORM: optical reflected microscopy; MALDI: matrix-assisted laser desorption/ionization mass spectrometry; for other abbreviations see footnote to Table 3, Table 4 and Table 8. Abbreviations: PGlu: poly(glutamic acid).

Compared to the grafting “from”, the positive side of the grafting “to” method is the use of presynthesized polymers, which can be synthesized by the methods of controlled polymerization with a narrow molecular weight distribution. In this case, immobilization of narrowly distributed polymers ensures uniform attachment, which ultimately provides a homogeneously modified cellulose filler. In turn, grafting “from” in some cases does not allow for controlling the polymer molecular weight and dispersity. At the same time, it represents a simpler in situ approach that requires less time and lower amounts of reagents.

In general, the functionalization of the cellulose surface with polymers provides a more considerable effect in terms of its hydrophobization and demonstrates more detectable improvements in the properties of composites (see Section 5). Moreover, both the properties of the filler and the composite material can be adjusted by varying the properties of the polymers used for modification.

### 4.4. Modification with Particles

The modification of cellulose micro- and nanomaterials is a relatively novel approach for influencing cellulose properties. In the current literature, one can find publications on the modification of the cellulose surface by inorganic nanoparticles (e.g., hydroxyapatite [232,233], silver [234,235], zinc-oxide nanoparticles [236]), and organic particles (e.g., latex) [237,238], as well as hybrid ones (e.g., organo-montmorillonite [239] and polydopamine-hydroxyapatite [240]).

In the last decade, interest in the development of biocomposites based on aliphatic polyesters containing cellulose micro- and nanomaterials modified with hydroxyapatite as a filler has attracted much attention. Such biocomposites are considered as scaffolds for bone regeneration. Aliphatic polyesters are hydrophobic and provide low cell adhesion and proliferation on their surface. In turn, introduction of cellulose can provide surface hydrophilicity and improve cell attractiveness. Hydroxyapatite (HA) is responsible for further scaffold biomineralization. Both cellulose particles/whiskers/fibers and hydroxyapatite can improve the mechanical properties of the scaffold. There are several ways to modify cellulose with hydroxyapatite. For example, Lu et al. reported the formation of hydroxyapatite on the surface of CNC in water from calcium-nitrate tetrahydrate and diammonium hydrogen phosphate at 70 ° C for 2 h and then additionally for 48 h at room temperature [232]. TEM analysis the resulting dispersion revealed an increase in the diameter of the cellulose nanocrystals due to adsorption of hydroxyapatite particles. Sridevi et al. separately prepared the yttrium substitute nano-hydroxyapatite and then used it to modify cellulose from rice husk, which was prefunctionalized with citric acid. The mixture of components was stirred for 24 h, ultrasonicated for 1 h, and dried at 80 °C to obtain dry composite powder [233]. The modification was evidenced by FTIR spectroscopy, TEM, and EDX analysis. The modification had almost no effect on the degree of crystallinity of the modified particles.

Li et al. used phosphorylated cellulose nanofibers to induce the growth of the hydroxyapatite particles on the CNF surface [241]. For this, phosphorylated CNF was incubated in the solution containing calcium chloride, sodium chloride, potassium chloride, magnesium chloride, and disodium hydrogen phosphate at 37 °C over 28 days. SEM analysis of treated CNF allowed the detection of only few spherical particles on the CNF surface after 7 days of incubation. More and bigger particles were found after 14 days, while after 21 and 28 days the particle sizes had increased considerably. The size and morphology of the HA particles varied greatly: spherical and cylindrical HA particles were detected on the CNF surface.

**Figure 14:**
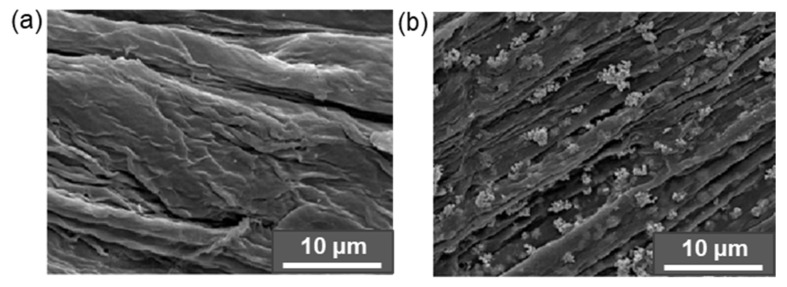
SEM images of neat CMC (**a**) and CMC modified with Ag nanoparticles (**b**). Reproduced from [245] with permission of Elsevier.

Silver nanoparticles are another of the most popular inorganic systems considered for the modification of cellulose materials. Silver nanoparticles are known to have antimicrobial properties, and their introduction into biomedical materials and food packaging is widely studied [242,243]. In the case of cellulose modification with the silver nanoparticles, the latter are obtained by the reduction of silver from silver nitrate. For instance, to modify CNW with Ag nanoparticles, Hasan et al. initially oxidized cellulose hydroxyls to carboxyl groups by TEMPO [244]. The formed carboxyl groups captured Ag ions from solution and were then reduced with sodium tetraborate during 1 h at 25 °C in the presence of 1wt% of CNC. The yield of silver in the product was 1.77 wt%. Different contrasts of CNW and Ag nanoparticles in TEM allowed their noncomplicated visualization and evaluation of the size of individual components. It was found that the CNWs with a length of about 200 nm and a diameter of 20–30 nm were covered by a large number of silver nanoparticles with the average size of about 5 nm. The analogous modification was carried by Lertprapaporn et al., who modified CMC with Ag nanoparticles but without the preoxidation of cellulose by TEMPO [245]. In this case, the yield of silver was reported to be 1.28%. The change in CMC morphology as a result of the modification with Ag nanoparticles is shown in Figure 14.

Recently, the electrostatic modification of CNF with various positively charged latex nanoparticles formed from amphiphilic copolymers has been reported [237,238]. Such an approach is also one of the possible ways to change the hydrophobicity of the cellulose surface in order to improve the interfacial adhesion to hydrophobic matrices. For this, poly(dimethylaminoethyl methcrylate)-based latexes with diameters from 79 to 146 nm were used to modify negatively charged cellulose surfaces prepared by CNF oxidation be TEMPO. The adsorption of the latex nanoparticles onto the surface of the CNF was confirmed by quartz-crystal microbalance with dissipation and atomic force microscopy. It was established that the total adsorbed mass increased from 10.5 to 44.4 mg/m^2^ with increased particle size from 42 to 96 nm. In addition, the increase in contact angle (up to 94°) and surface roughness (AFM) also testified the latex adsorption [238].

## 5. Aliphatic Polyesters/Cellulose Composites

### 5.1. Preparation of Aliphatic Polyesters/Cellulose Composites

**Figure 15:**
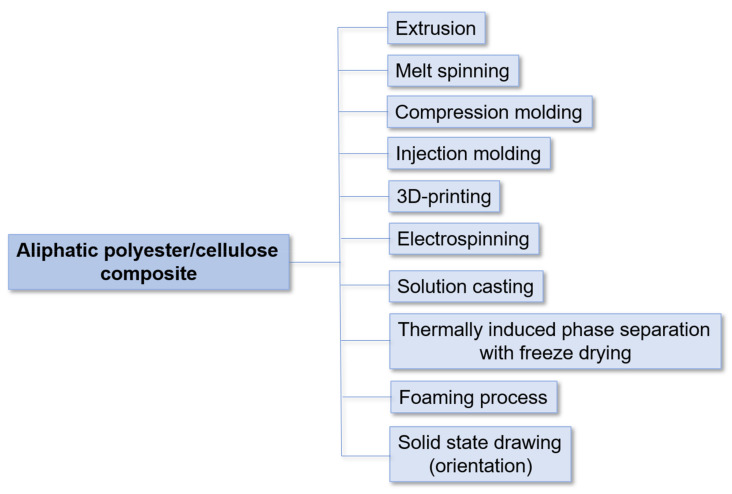
Scheme for different techniques of composite preparation.

The effective dispersion of cellulose in the polymer matrix is a key task for producing the homogeneous biocomposites based on hydrophobic aliphatic polyesters and cellulose micro- or nanomaterials [39,45]. Hot blending (direct and continuous melt mixing) or mixing an aliphatic polyester solution with dry/suspended cellulose is generally used to obtain composite blends. In terms of producing aliphatic polyester/cellulose composites on an industrial scale, hot blending is of most interest [39]. Various kinds of equipment, such as an internal mixer or a single/two-screw extruder, were used to optimize this approach with respect to composite components [2,39]. In spite of the applied shear/elongational forces during this type of mixing, the hydrophilicity and strong interchain interactions of cellulose, as well as high temperatures of processing, can lead to heterogeneous material as well as thermal degradation of cellulose or aliphatic polyester [39,79,90,92]. In this regard, various methods have been proposed to improve the distribution of cellulose in the polymer matrix: the main ones are the modification of cellulose, polyesters, and the use of additives (surfactants, compatibilizers) [2,39]. An alternative method of obtaining a blend that avoids thermal degradation of the components is the approach of dispersing cellulose in an aliphatic polyester solution, followed by evaporation of the solvent to obtain the material. The disadvantages of this method include a limited set of suitable solvents due to the possibility of removing them without the use of high temperatures, the solubility of aliphatic polyesters, and increased aggregation of cellulose chains/particles [39]. Subsequent application of such techniques as melt compounding (extrusion, melt spinning, compression molding, injection molding, 3D printing), solution casting, electrospinning, etc. to the resulting blends allows the fabrication of aliphatic polyester/cellulose composites of various shapes [2,39,65]. Types of techniques for the fabrication of composite materials based on cellulose and aliphatic polyesters are summarized in Figure 15.

Extrusion can be implemented both in the laboratory and on an industrial scale, and allows for the pushing of the melt through the extruder die to obtain composite material of different geometries, often in the form of pellets and filament [2,65]. The melt-spinning technique is based on the formation of composite fibers using the melt-filament technique from melt-extruded pellets. The composite sheets can be produced by the compression-molding technique, which is based on the pressing of blends or layered components under certain temperatures and pressures. It allows for the introduction of a large quantity of cellulose materials with high efficiency (up to 70%) [246]. Injection molding is the injection of pressurized samples into a special preheated mold followed by curing. The temperature and holding time can be varied and require optimization.

3D-printing techniques make it possible to produce products of different—including complex—shapes with high accuracy and reproducibility. The greatest potential for obtaining polymer cellulose-based composites from the melt is the fused-deposition-modeling (FDM) type of 3D printing, which consists in nozzle-deposition-based extrusion. This method allows the production of three-dimensional biodegradable materials for a wide variety of fields (food packaging, construction and automobile industry, and biomedicine) [2,4,65]. 

Using the solution-casting method, aliphatic polyester/cellulose composites can be formed as films. This method involves casting a suspension of cellulose in an aliphatic polyester solution onto a flat substrate, followed by evaporation of the solvent in the first step in air at room temperature, and then sometimes using vacuum drying and/or slight heating (up to 60 °C) [39,173,231]. The main obstacle of this method is the impossibility of producing composites on an industrial scale. Furthermore, composites from a filler suspension in polymer solution can be fabricated as supermacroporous products using the method of thermally induced phase separation followed by freeze drying [2,4]. 

Electrospinning (also referred to as Electrostatic fiber spinning [51]) is applicable to both polymer melts and solutions and allows the formation of composite polyester/cellulose fibers from 100 nm to several micrometers thick under electrostatic forces [39,247]. The fiber mats obtained with this technique are characterized by a large surface area and are of great interest for tissue engineering, drug delivery, cosmetics, sensors, conducting nanofibers, etc. [247]. The electrospinning method is economical and performed under ambient conditions. The application of solid-state drawing against aliphatic polyester/cellulose-based composite films using a tensile tester with the ability to heat and thermostat the sample has also been reported. This approach led to an efficient orientation of the filler and polymer, and as a consequence, to the formation of an organized molecular structure with improved mechanical and thermal properties by increasing the crystallinity of the material [2].

In addition, foam composites based on aliphatic esters and cellulose micro-/nanomaterials can be produced, but the implementation of the foaming process is difficult at low melt strength [39]. Recently, hybrid approaches to obtain composites based on aliphatic polyesters and cellulose by combining different types of mixing (“wet” and “dry”) and molding have been increasingly utilized [2,39]. This is primarily due to the tendency of cellulose to aggregate, which makes it difficult to disperse homogenously in the polymer matrix. The use of predried cellulose, which is prone to irreversible aggregation, makes it especially difficult to obtain a homogeneous composite mixture. Thus, attempts are made to avoid the pulp-drying stage [51]. Typically, cellulose and polymer-based masterbatches are resorted to (casting from solution, in situ polymerization, centrifugation), followed by dilution with aliphatic polyester by melt blending. There have also been reports on the introduction of nanocellulose suspension into the extruder (liquid-assisted feeding) [39]. A combination of solvent casting and extrusion has been reported to prevent thermal degradation and achieve good dispersibility of the cellulose material [2].

Processing type has a great influence on the properties of the obtained composites. When choosing one or the other approach to obtain aliphatic polyester/cellulose composite, the characteristics and properties (thermal resistance, dispersibility, solubility) of both cellulose and polymer matrix used, as well as the desired final design of the material, should be considered [39,45,51].

### 5.2. Effect of Cellulose Modification on Mechanical Properties of Composites

The production of biocomposites from aliphatic polyesters and cellulose micro- and nanomaterials has attracted much attention due to the prospects of using such materials for biomedicine, food packaging, and other technical biodegradable materials [105,248,249,250,251]. However, the high hydrophilicity of cellulose, and in turn, the high hydrophobicity of aliphatic polyesters makes it difficult to obtain homogeneous composites with good mechanical characteristics. A decrease in the tensile properties of composites using neat cellulose has been observed by several research groups [231,252,253,254]. For instance, Liu et al. reported a decrease in tensile strength of PLA (60 MPa) after its filling with neat cellulose fibers [252]. The decrease from 52 to 42 MPa was observed when the filler content was increased from 3 to 15 wt%. Moreover, the tensile strength depended on the 3D-printing method. The strongest composite was obtained when parallel printing was performed.

Table 10 illustrates a summary of the tensile properties for aliphatic ester/cellulose composites for selected papers. In the case of adsorption and covalent modification of cellulose materials with small molecules, the mechanical properties differ markedly in different studies. Only a slight improvement was observed for composites obtained with cellulose micro- and nanomaterials modified by adsorption [114,120,123,125]. A decrease in tensile modulus, tensile strength, and yield stress was observed when comparing PLA-based composites prepared by electrospinning and filled with neat CNC and CNC modified by adsorption with ethoxylated nonylphenol phosphate ester [122]. In this case, the low effect of modification on the mechanical properties of composites can be partially explained by the possible desorption of small molecules from the surface of cellulose during the production of the composites.

**Figure 16:**
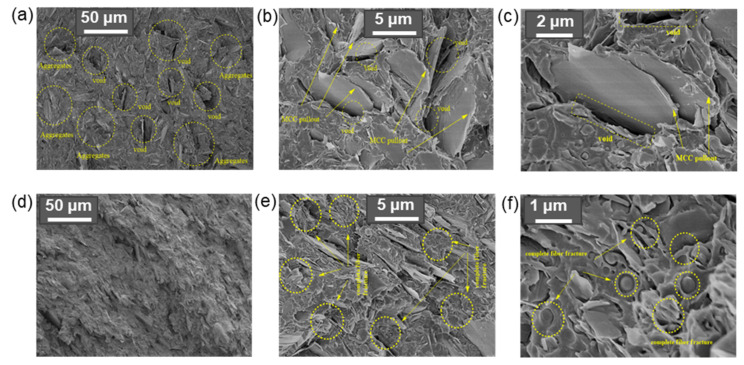
Distribution of neat and modified CMC (3 wt%) in the PLA matrix (SEM): (**a**) surface of PLA/CMC; (**b**,**c**) fractured surface of PLA/CMC at different magnifications; (**d**) surface of PLA/modified CMC; (**e**,**f**) fractured surface of PLA/modified CMC at different magnifications. The CMC modification with toluene-2,4-diisocyanate was carried out for 24 h to reach the maximum functionalization. Reproduced from [32] with permission of American Chemical Society.

Xu et al. revealed a stronger interfacial adhesion between PLA and CNC covalently modified by acetic acid [140]. As a result, the composite films prepared by solution casting showed higher strength and Young’s modulus than ones with neat CNC by about 20%. The improvement in tensile strength and Young’s modulus by 38 and 71%, respectively, for PLA filled with hydrophobized MFC due to its transesterification with vinyl-laurate groups (compression-molded composites) was also observed by Li et al. [148]. At the same time, modification of CNF with fatty acids by transesterification and production of composites with PLA-PBS by combination of extrusion and molding showed no significant effect on the tensile strength and Young’s modulus when 1 and 3 wt% filler were used [42]. Moreover, the parameters obtained were very close to those obtained for unmodified cellulose. Only the composite containing a 5 wt% filler showed an 25% increase in tensile strength. An improvement in tensile properties of not more than 13% for composites prepared by solution casting and based on PLA filled with bacterial cellulose fibers modified with citric acid was found by Ramirez et al. [132]. Practically no effect on the mechanical properties of PLA-based composites (also prepared by solution casting) was observed when CMC was modified with palmitic-acid residues from olive oil [146]. The modification of cellulose fibers with formic acid and variation of the content of formyl groups from 1.7 to 15.8% provided an increase in tensile strength of the PLA-based composites produced by solution casting [137]. Even at the lowest formyl group content on the cellulose surface, the tensile strength and Young’s modulus increased by more than 200%. At the same time, these parameters for a PLA composite filled with 1 wt% cellulose fiber containing 15.8% formyl groups increased by more than 300%. Such different results can be related to the properties of both the matrix polymer and the filler. For the latter, the source of the cellulose, the effectiveness of its modification, and the content in the matrix can lead to differences in composite properties. As illustration, the morphology of PLA composites filled with neat CMC and CMC modified with toluene-2,4-diisocyanate is presented in Figure 16.

Gwon et al. compared three different commercial PLAs (4032D, 3001D, and 2003D, Ingeo, Natureworks LLC, Blair, NE, USA) for preparing composites with CNC modified with toluene-2,4-diisocyanate [187]. In all cases, the use of modified CNC showed higher tensile-strength values than the use of the unmodified filler. The best mechanical properties were observed for composites prepared by solution casting and based on PLA with higher molecular weight and crystallinity (4032D), while composites based on PLA with low molecular weight and high crystallinity (3001D) or low crystallinity and high molecular weight (2003D) showed reduced tensile properties.

**Figure 17:**
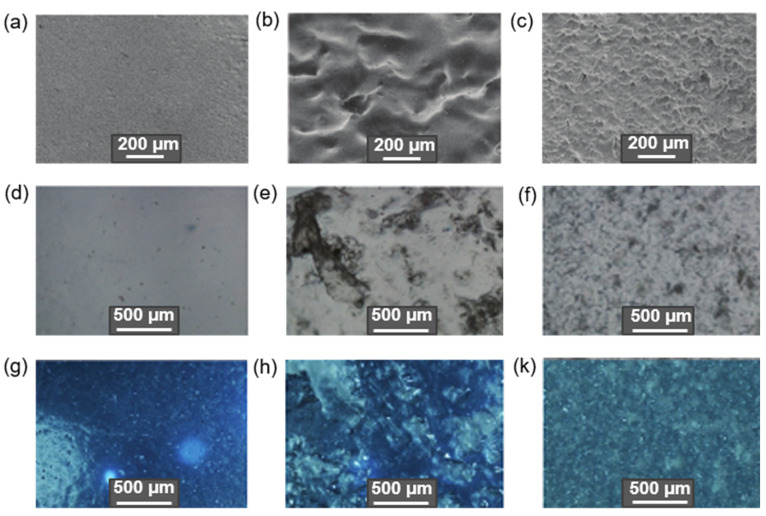
Images obtained by SEM (**a**–**c**), optical (gray) (**d**–**f**) and reflected (blue) (**g**–**k**) microscopy for pure PLLA (**a**,**d**,**g**), and its composites with neat CNC (15 wt%) (**b**,**e**,**h**), and CNC modified with poly(glutamic acid) (15 wt%) (**c**,**f**,**k**) (reproduced from [230] under the terms of the Creative Commons CC BY license).

Recently, Voronova et al. investigated the effect of the content of CNC with adsorbed PVP in the PCL matrix on the tensile properties of composite films prepared by solution casting [127]. Increasing the filler content from 5 to 15 wt% resulted in a twofold increase in Young’s modulus, but at the same time a threefold decrease in tensile strength. A similar tendency was also observed for the composite films fabricated by solution casting when CNC was grafted with poly(glutamic acid) and used as a filler to PCL and PLA [230,231]. The effect on changes in composite morphology and material homogeneity depending on the used filler is demonstrated in Figure 17.

**Figure 18:**
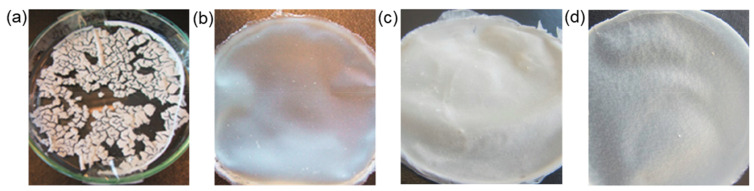
Images of PHB composite films: (**a**) filled with 5 wt% of neat CNC, (**b**) filled with 5 wt% of CNC with adsorbed surfactant, (**c**) filled with 20 wt% of CNC with adsorbed surfactant, and (**d**) filled with 20 wt% of CNC grafted with poly(lactic acid). Reproduced from [203] with permission of Elsevier.

The effect of cellulose-surface grafting with aliphatic polyesters for enhancing the filler compatibility with PLA, PCL, or PLGA matrices have been studied in several papers [67,201,208,213]. Two main trends can be found for composite films prepared by solution casting: an increase in tensile modulus with a simultaneous decrease in elongation at break and vice versa. For example, Chai et al. varied the content of CNC grafted with PDLA in the PLA matrix from 1 to 10 wt % and found that the best mechanical properties were observed with a filler content of 2 wt% [201]. In this case, an increase in Young’s modulus and tensile strength of about 30% was established, while elongation at break was reduced by 34%. A similar trend, but with a sharper decrease in elongation at break, was also observed by Averianov et al. for PCL-based composites filled with CNC-g-PLLA [208] and by Habibi et al. for PCL-based composites filled with CNC-g-PCL [67]. In turn, in the case of PLGA composites filled with CNC-g-PLLA, there was a 17–50% decrease in Young’s modulus and a 20–50% increase in elongation at break, depending on the filler content [203]. In the case of using CNC grafted with PLLA as a filler to PHB, a significant reduction in the brittleness of the matrix polymer was observed (Figure 18).

Thus, in general, hydrophobization of the cellulose surface improves the compatibility of modified cellulose with aliphatic polyesters, which contributes to enhanced mechanical properties. However, the approach to cellulose modification, its efficiency, filler content, composite fabrication technique, as well as aliphatic polyester characteristics affect the properties of the resulting composite material.

### 5.3. Effect of Cellulose Modification on Thermal Properties and Crystallization

The thermal stability of composites is one of the key properties when considering the thermoplastic-processing techniques. In general, the aliphatic polyesters filled with unmodified cellulose micro- and nanomaterials or modified with small molecules exhibit reduced thermal stability [142,171,255]. For example, Hong et al. observed the decrease in thermal stability (TGA) of composite fabricated by injection molding in comparison with pure PLA [255]. In particular, a decrease in onset temperature from 345.2 °C for PLA to 306.8 °C for the PLA filled with the 30 wt% of silanized bagasse fibers (40–50%cellulose, 25–35% hemicellulose, and 15–35% lignin) was detected. In turn, the temperature of a maximum mass loss for pure PLA and its composite with silanized fibers was reduced from 363.7 to 331.4 °C. A similar trend was established by Li et al. who detected a reduction in the onset temperature from 344.2 °C for pure PLA to 246.4 °C for its composite prepared by solution casting with the use of silanized CMC as a filler and acetyl tributyl citrate as a plasticizer [171]. By preparing composites based on PHB with acetylated CMC, Ribero et al. demonstrated that a slight improvement in thermal stability can be observed if the filler content does not exceed 0.5 wt%. In turn, the onset and endset temperatures decreased at 0.75 wt% of the filler [142]. Kasa et al. varied the content of the neat and acetylated CNC from 1 to 7 wt% in the PLA matrix [31]. The highest degradation temperature (325 °C) was detected for composite obtained by the casting of PLA solution containing 1 wt% of acetylated CNC. The degradation temperatures for PLA/neat CNC composite (1 wt%) and PLA were lower by about 20 and 35 °C, respectively. Increasing the filler content to 3 wt% contributed to a temperature decrease of 5 °C and then remained unchanged when the filler content was increased to 7 wt%.

In contrast to the modification with small molecules, modification of cellulose by grafting with aliphatic polyester can improve its thermostability. Recently, Simao et al. compared the thermostability of unmodified cellulose nanowhiskers with these grafted with PCL and found that the maximum mass loss was observed at about 300 °C for the neat CNW and 370 °C for the CNW-g-PCL [211]. As a result, the onset temperature of degradation of the PCL/PBSA (30/70) composites produced by electrospinning and containing 1 and 5 wt% of CNW-g-PCL remained at the same level as for pure aliphatic polyesters (295 and 294 °C, respectively). The maximum degradation temperature was reduced after filling PCL/PBSA with CNW-g-PCL, but not as dramatically as for modification with small molecules. These temperatures were 441, 435, and 438 for nonfilled polyester blend and filled with 1 and 5 wt% CNW-g-PCL, respectively. The same tendency was observed for PLA filled with CNC-g-PLA (composites fabricated by electrospinning). A neat PLA started to degrade at 285 °C and degraded until 390 °C [213]. With the addition of CNC-g-PLA (3–7 wt%), the initial degradation temperature was elevated to 300 °C and the final degradation temperature reached 400 °C.

DSC analysis of pure aliphatic polyesters and their composites with cellulose micro- and nanomaterials allowed the conclusion that the glass-transition temperature (*T_g_*) and melting temperature are reduced for composites with neat cellulose or modified with small molecules. For example, Way et al. observed a decrease in glass-transition temperature from 60.4 °C for pure PLA to 56.5, 56.6, and 54.8 °C for its composite with neat, silanized, and acetylated lignincellulose fibers (30 wt%), respectively [184]. At the same time, the melting temperatures were varied within 1 °C.

Simao et al. studied the thermal properties of PCL/PBSA mixture and its composite with CNW-g-PCL produced by electrospinning [211]. They found that the melting temperature for the 50/50 blend of two aliphatic polyesters was 64 °C. The introduction of 1 and 5 wt% of cellulose nanowhiskers grafted with PCL led to a decrease in melting temperature to 63 and 60 °C. At the same, an increase in crystallization temperature from 24 °C for polymer mixture to 29 and 35 °C for the 1 and 5 wt% composites was detected. In turn, a different trend in the glass-transition temperature was observed for PLA composites with CNC-g-PLA. In this, case *T_g_* increased with increasing filler content from 54.9 for pure PLA to 57.1 °C for composite containing 7 wt% of the filler. Similar to the grafting of cellulose with polymers, the modification with fatty acids also favored the improvement of properties for PLA-based composites fabricated by solution casting [136]. For example, modifying CMF with oleic acid and using it as a filler (12 wt%) in PLA provided an increase in the melting temperature from 125.5 to 159.2 °C.

**Figure 19:**
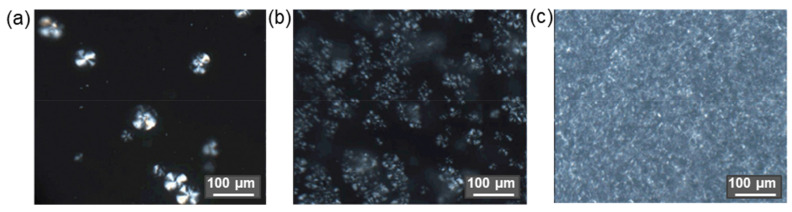
Polarized optical microscopy of PLLA crystallization at 120 °C after heating to 185 °C and hold for 4 min: PLLA (**a**), PLLA with 10 wt% CNC-g-PLLA (**b**) and PLLA with 10 wt% CNC-g-PDLA (**c**) (reproduced from [201] with permission of Elsevier).

Fang et al. found that during the melt-crystallization process, CNC-g-PLLA provided better nucleation and less restriction to chain mobility than CNC-g-PDLA at undercooled conditions [205]. In particular, no melt crystallization was detected in neat PLLA during the cooling process, while the melting-crystallization temperatures were 92.3 and 97.8 °C for 5 and 15 wt% composites produced by injection molding, respectively. In turn, no melt crystallization was observed when PLLA was filled with 5 wt% of CNC-g-PDLA, whereas increasing the filler content to 15 wt% revealed a melt-crystallization peak at 92.5 °C. Almasi et al. compared the PLA crystallization capability with its composites with modified CMC prepared by solution casting. A broad crystallization peak was observed by DSC at 57.5 °C [136]. In turn, the composites containing 4 and 8 wt% of CMC modified with oleic acid demonstrated the enhanced crystallizability (about 75 °C). At the same time, Chai et al. revealed that CNC-g-PDLA increased the crystallization ability of the PLLA matrix more than CNC-g-PLLA (Figure 19) [201].

### 5.4. Effect of Cellulose Modification on Composite Degradation

Degradation of materials fabricated from aliphatic polyesters occurs heterogeneously as a result of hydrolysis of ester bonds under the action of water and can occur both in presence or absence of enzymes [80,84,86,98]. Degradation of all aliphatic polyesters follows the same principle. Usually, the degradation starts from a nonenzymatic decrease in molecular weight, and then the enzymatically catalyzed hydrolysis in the case of degradation in the body is joined. The biodegradation of aliphatic polyesters is catalyzed by enzymes with esterase activity [86,256,257]. Some enzymes, such as proteinase K, for example, can cleave the core molecule itself, not just its oligomeric products preproduced during hydrolysis [8]. In the environment, the biodegradation is provided by the metabolism by certain microorganisms to CO_2_ and H_2_O [85]. In all cases, initially the hydrolytic cleavage of ester bonds occurs in the amorphous sites and then in the crystalline zones [8,85]. Thus, the amorphous PLA is degraded much faster than other semicrystalline aliphatic polyesters.

The rate of degradation is determined by molecular weight, crystallinity, and degradation conditions [89,98]. Polymers with higher molecular weight take a longer time to biodegrade because more ester bonds need to be cleaved to form water-soluble fragments [98]. The degradation rate of aliphatic polyesters is affected by such factors as temperature, the ability of water to penetrate the polymer matrix, material thickness and morphology, hydrolysis-product removal, pH, presence of catalysts, UV, moisture, etc. [5,8,84,85,86,93]. Aliphatic polyesters have been shown to dissociate very rapidly in strongly acidic and strongly basic environments. Under composting conditions, when the medium temperature can reach 70 °C, the degradation rate can increase significantly [8]. UV exposure also accelerates polymer degradation [85].

As a main product formed during aliphatic-ester degradation, a corresponding carboxylic acid is released, namely lactic acid, glycolic acid, caproic acid, 3-hydroxybutyric acid, succinic acid, etc. In turn, the release of carboxylic acid(s) affects the pH of the surrounding area towards acidification. In vivo, such local acidification favors the appearance of inflammatory reactions in tissues. Therefore, the faster the material destruction rate and the worse the tissue can neutralize this effect, the more inflammation can develop in the implant area [98].

The degradation rate for aliphatic polyesters of similar molecular weight and under similar conditions can be described by the following range: PGA > PLGA ≥ PDLLA > PLLA > PHB > PCL > PBS (Table 2). PGA is characterized by a very rapid biodegradation. For instance, in vitro degradation of commercial PGA-based sutures (Dexon^®^) approved by the FDA in 1969 resulted in a 42% decrease in polymer weight and loss of mechanical properties in 28 days [86]. PLGA and PLA are considered as polymers with moderate degradation rates while PCL and PBS are slowly degradable aliphatic polyesters [80,90,258]. In vitro experiments in various media (composting, burial in soil, seawater, presence of lipase enzyme or activated sludge) demonstrated that PBS biodegradation was significantly lower than for PCL and PHBV but higher than for petrochemical plastics [91]. It is known that microorganisms such as *Fusarium solani* can contribute to the degradation of PBS.

The effect of the cellulose as filler to aliphatic polyesters on the degradation rates of biocomposites have been studied in several papers [171,234,237,251,259,260]. An investigation of enzymatically catalyzed (proteinase K) degradation of PLA composites with neat CNC and CNC modified by adsorption with acid phosphate ester of ethoxylated nonylphenol (surfactant Beycostat A B09) showed a lower weight loss for composites prepared with modified CNC via solution-casting technique [259]. After three days of incubation of the films, the weight loss for pure PLA and PLA filled with neat CNC approached 100%. At the same time, the PLA composites with modified CNC under the same conditions lost only about 32% of their weight within a week. Furthermore, increasing the filler content in PLA from 1 to 3 wt% significantly slowed down the weight loss of the material. While the material containing 1 wt% filler completely degraded within 9 days, the composite filled with 3 wt% modified CNC demonstrated an 85% weight loss within 21 days. Examination of these composites by SEM revealed a change in the morphologies of PLA and PLA/CNC composites after just 2 h of incubation with enzyme. Surface erosion with holes and channels was observed. In turn, PLAs filled with modified CNC retained their topography even after 24 h of incubation in the medium containing proteinase K. Analysis of the polymer crystallinity supported the tendency known for the degradation of pure aliphatic polyesters: in the presence of the enzyme, amorphous regions were destroyed first in respect to crystalline ones. The higher values of crystallinity degrees were detected during the different degradation times.

Vilela et al. investigated the enzymatic degradation of composites produced by injection molding of PCL with neat CNF and CNF electrostatically covered with polymethcarylate-based latexes in presence of lipase over 2.5 months [237]. The highest weight loss was detected for the PCL/CNF composite, while the lowest one was found for pure PCL. The PCL composite containing CNF modified with latexes showed an intermediate weight loss.

A study of the degradation of PLA films and their composites fabricated by solution casting with the use of unmodified CNW and CNW modified by adsorption of PEG monooleate, PEG-300 or PEG-1000 in garden soil confirmed the effect of microorganisms on the substrates [260]. The authors found that CNW-surface modification with PEGs accelerated film disintegration after incubation for 90, 120, and 150 days in soil at 29 °C and 28% soil moisture. The highest disintegration rate was detected when PEG-1000 was used as a modifier. A similar soil-burial experiment was performed by Li et al. for PLA composite films filled with neat CMC and CMC silanized by APTES and prepared by solution casting [171]. It was found that adding CMC contributed to the higher degradation rate of the films. During the degradation process, the films partly lost their transparency, and the color became yellow and the surface wrinkled. After 60 days of incubation, 98%, 96%, and 88% of the original mass were maintained by PLA, PLA/CMC-APTES, and PLA/CMC, respectively. After 90 days, the most pronounced degradation was found for the PLA/CMC composite (~26% weight loss), while the least degradation was found for the pure PLA material (around 7% weight loss). The covalent modification of CMC with APTES favored a slower degradation rate (20% weight loss) compared to the unmodified material, but it was still evident compared to pure PLA.

Thus, the introduction of the unmodified natural fibers into the aliphatic polyesters accelerated their degradation in vitro [80,91,237,251,259]. At the same time, in the case of cellulose modification, different trends were observed that may be connected with the nature of the modifier and the method of modification (adsorption or covalent binding).

### 5.5. Effect of Cellulose Modification on Biological Properties

Being biocompatible and hydrophilic, cellulose is widely considered not only for production of degradable packaging [261] and technical materials [158,245,262], but also for the development of biomedical materials [263]. The biocompatibility of cellulose materials in vivo and in vitro is supported by several studies [230,231,249]. For example, Codreanu et al. evaluated the viability and cell-proliferation potential for the composites prepared by melt mixing of PHB with bacterial cellulose using the mouse preadipocyte (3T3-L1) cell line [249]. Pure PHB was used as a control material. The MTT assay performed after 24 h and 5 days of incubation revealed no difference between composites containing 1 and 2 wt% of cellulose filler and pure PHB. The macroporous scaffolds produced from pure PHB and its composites with bacterial cellulose were tested in vivo for bone regeneration over 4 and 20 weeks. The enhanced osteogenic differentiation was estimated for the composite materials compared to pure PHB. The best regeneration potential was detected for PHB composite containing 2 wt% of bacterial cellulose.

**Figure 20:**
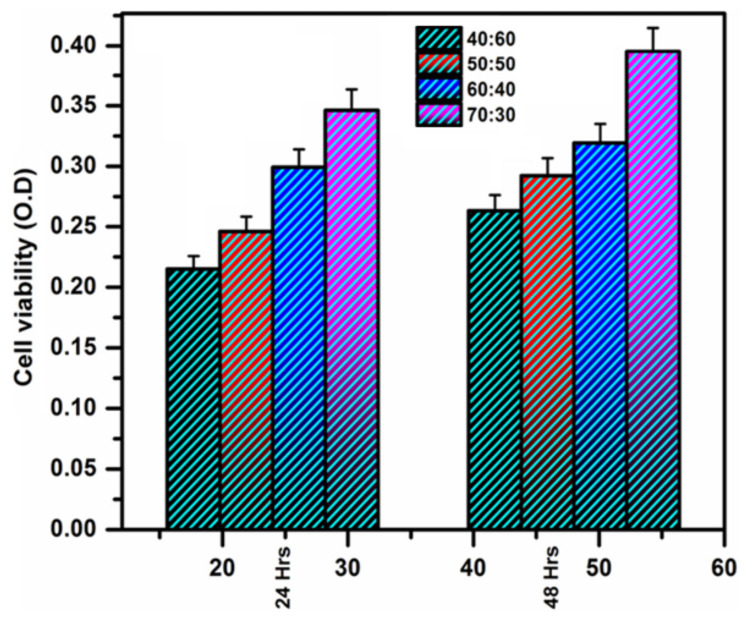
Cell-proliferation study (MG-63 cell line) for the PLA/CNF-HAP electrospun composites of different compositions reproduced from [264] with permission of John Wiley & Sons).

Taking into account that modification of cellulose micro- and nanomaterials can change surface properties, biological testing of modified cellulose should be performed to confirm the biocompatibility of a potential biomaterial. The proliferation of osteosarcoma cell lines (MG-63) during 48 h at the surface of PLA electrospun-fiber composites containing cellulose nanofibers modified with hydroxyapatite (HAP) particles was recently reported [264]. The highest cell growth was observed when using the PLA/CNF-HAP composite with a 70/30 component ratio (Figure 20).

**Figure 21:**
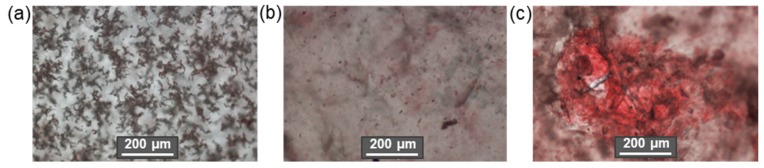
In vitro mineralization study by optical microscopy of pure PCL (**a**) and its composites with 15 wt% neat CNC (**b**) and 15 wt% CNC modified with PGlu (**c**). Staining was performed with alizarin red S. Red color indicates the presence of calcium deposits (reproduced from [224] under the terms of the Creative Commons CC BY license).

The evaluation of cell viability on the surface of PLLA, PDLLA, or PCL composite films produced by solution casting and containing 5, 10, or 15 wt% of neat CNC or CNC modified with poly(glutamic acid) revealed the similar viability of rabbit mesenchymal stem cells after 24 h [230,231]. The biocompatibility of the composites was assessed after a 1-month in vivo subcutaneous biocompatibility test in rats. All composites demonstrated higher compatibility than pure PDLLA or PCL, which can be related to the partial surface hydrophilization due to the addition of CNC. However, the composites filled with CNC-PGlu caused the formation of a thinner fibrous capsule and less inflammation. This fact can be explained by the lower roughness of the composite due to better distribution of the modified CNC in the polyester matrix. The PCL-and PLLA-based composites, compared to PDLLA ones, demonstrated less inflammation due to slower hydrolysis and less acidification. Moreover, modification of CNC with PGlu improved the mineralization of the composites (Figure 21), which makes these materials promising for the use as scaffolds for bone-tissue regeneration.

Recently, the hemolytic activity of CMC modified with poly(2-hydroxyethyl methacrylate), poly(glycidyl methacrylate) (PGMA), and poly(lauryl methacrylate-co-methyl methacrylate) was evaluated by Rabbi et al. [265]. Incubation of modified CMC in human blood was performed for 1–6 h at 37 °C, varying the concentration from 0.25 to 1.00 mg/mL. In all cases, the degree of hemolysis did not exceed 1%, despite the hydrophobization of the cellulose surface with polymethacrylates. At the same time, CMC-PGMA showed twice as much hemolytic activity (about 1%) as other CMC types.

Fortunati et al. studied the antibacterial activities of composites fabricated by solution casting from PLA and neat CNC or CNC covered with surfactant Beycostat A B09 with or without Ag nanoparticles against *S. Aureus* and *E. coli* [121,234]. All composites showed an antibacterial activity higher than pure PLA, but as it was expected, composites containing Ag nanoparticles demonstrated more pronounced antibacterial activity.

From the presented few works that have reported on the study of the biological properties of modified cellulose, it is obvious that targeted modification of cellulose with appropriate components provides an improvement of the properties of interest.

## 6. Conclusions and Future Outlook

In this review, the different approaches to the modification of the surface of cellulose micro- and nanomaterials have been summarized and discussed. In general, the surface hydrophobization positively influences the interfacial compatibility between cellulose and aliphatic polyesters. It in turn contributes to the enhancement of mechanical and thermal properties, and improves barrier properties and stability towards degradation in comparison with composites with neat cellulose. However, a degree of these improvements depends on the nature of the modifying agent, the method of modification, the efficiency of modification, and the filler content in the matrix of aliphatic polyester. Obviously, the covalent modification of the cellulose provides more stable linking. The optimal content of the filler is in the range from 1 to 10 wt%. Polymers and fatty acids provide higher impact on the properties of the final composite materials than smaller and more hydrophilic molecules. Furthermore, the characteristics of cellulose, its source and premodification treatment also play an important role since they determine the crystallinity of the sample, surface functionality, degree of purity, and dimensions.

Currently, one of the most-used methods for composite preparation is solution casting. However, this method is not suitable for large-scale production, and also has such drawbacks as retention of solvent traces and possible aggregation and precipitation of filler during solvent evaporation. Hot pressing, melt blending through extrusion, and injection or blow molding are more technologically advanced and better-controlled techniques that have industrial application prospects. Such parameters as crystallization, orientation, dispersion, and distribution of cellulose fillers in the melt-polymer matrix should be controlled when fabricating the composite products.

In general, both neat and modified cellulose-containing composites of aliphatic polyesters demonstrate high biocompatibility in vitro and in vivo. Given that many aliphatic polyesters as well as cellulose are approved for biomedical applications, the introduction of such composites into biomedical practice is very likely in the future.

## Figures and Tables

**Table 1:** Cellulose micro- and nanomaterials.

Type	Fabrication [26,28,39,44]	Structure [26,27,39,46]	Size [26,27,28,46,69,70]
CMF	Mechanical treatment	Long thin flexible aggregates of elementary fibrils/microfibrils with amorphous and crystalline domains	Width 20–100 nm Length 0.5—several μm
CNF	Mechanical with/without chemical and/or enzymatic treatment	Long thin flexible structures with amorphous and crystalline domains	Width 2–100 nm Length 0.5—several μm
CMC	Hydrolysis with diluted inorganic acids with/without mechanical treatment	Rigid crystalline spherical or rod-shaped particles (large aggregates of nanocrystals)	10–200 μm
CNC	Hydrolysis with concentrated inorganic acids with mechanical and/or ultrasound treatment	Rigid whiskers, needle-like crystalline particles	Width 3–50 nm Length 100–500 nm (up to several μm for cellulose from algae, tunicate and BC)

Abbreviations: CNC: cellulose nanocrystals (nanocrystalline cellulose); CMC: cellulose microcrystals (microcrystalline cellulose); CNF: cellulose nanofibers; CMF: cellulose microfibers.

**Table 2:** Some characteristics of commonly used aliphatic polyesters.

Characteristics	PLLA	PDLA	PDLLA	PGA	PHB	PCL	PBS
Crystallinity, (%)	Up to 40 (50, thermal treatment) [3,11,84,85]		Amorphous [11,84]	45–77 [86]	50–80 [79,87,88]	Up to 69 (up to 80 during degradation) [89,90]	35–45 [80,91]
Density, (g/cm^3^)	1.29 [84]	1.25 [84]	1.25 [84]	1.50–1.71 [86,92]	1.26 [88]	1.07–1.20 [89]	1.23–1.26 [80,91]
*T_g_*, (^o^C)	55–80 [84]	40–50 [84]	43–60 [11,84]	35–40 [86,93]	4–9 [79,87]	(−65)–(−54) [89,93]	(−45)–(−10) [91]
*T_m_*, (^o^C)	170–200 [11,79,84]	120–150 [84]	120–170 [84]	220–230 [86,93]	165–185 [79,87]	55–70 [90,93,94]	90–120 [91]
E, (GPa)	2–4 [93]		1–3.5 [93]	6–7 [86,93]	2.5–3.5 [79,87,88]	0.21–0.44 [89,90]	0.03–0.71 [80]
Tensile strength, (MPa)	60–70 [93]		40 [93]	60–110 [86]	20–43 [79,87,88]	4–785 [89,90]	20–35 [80,91]
Elongation at break, (%)	2–6 [93]		1–2 [93]	1–20 [86,93]	5–10 [79,88]	20–4000 [89,93]	560 [91]
Solubility	CHCl_3_, 1,4-dioxan, furan [84]	Ethyl lactate, ethyl acetate, THF, DMF, DMSO, xylene [84]	Aceton, CDCl_3_, 1,4-dioxan, furan [84]	Hexafluoro-isopropanol (only for polymers with M ˂ 45,000) [86,93]	Hot CHCl_3_ and CH_2_Cl_2_ [87]	1,4-dioxane, 2-nitropropane,cyclohexanone, THF, toluene, benzene, CHCl_3_, CCl_4_, CH_2_Cl_2_ [90]	CHCl_3_ [95]
Degradation	More than 2 years [93]		3–6 months [93]	From 6 weeks to 6 months [86,93,96]	4–12 weeks [97]	From several months to several years [89,98]	1–30% for 6 months [99,100]

Abbreviations: THF: tetrahydrofuran; DMF: dimethylformamide; DMSO: dimethyl sulfoxide. Physical quantities: *T_g_—*glass-transition temperature; *T_m_—*melting temperature; *E—*elastic modulus.

**Table 7:** Summary on covalent modification of cellulose micro- and nanomaterials with alkyl/aryl isocyanates for the preparation of composites with aliphatic polyesters.

Type of Cellulose	Modifying Agent(s)	Filler Content (wt%)	Matrix Aliphatic Polyester	Processing/ Design of Composites	Characterization Methods	Refs.
CNF and CMF	*n*-Octadecyl isocyanate	3–12	PCL	Casting/Films	TEM, SEM, DSC, DMA and tensile tests	[185]
CNC	*n*-Octadecyl isocyanate	5 or 10	PCL	Casting/Films	FTIR, XPS, SEM, TGA, rheological and wettability tests	[29]
CMC	Toluene-2,4- diisocyanate	1–5	PLA	Extrusion + Molding/ Films	Elemental analysis, FTIR, SEM, wettability and mechanical tests	[32]
CNC	Toluene-2,4- diisocyanate	1–5; 1–9	PLA	Casting/Films	ATR-FTIR, UVis, TEM, AFM, TGA, tensile tests; NMR, SEM, XRD, DSC	[186,187]
CNC	Isophorone diisocyanate	1 or 5	PLA	Casting/Films	Elemental analysis, NMR, XRD, SEM, wettability and mechanical tests	[188]
Holocellulose powder	4,4′-Methylenebis(phenyl isocyanate)	5–30	PBS	Hot pressing/Sheets	FTIR, SEM, wettability, water adsorption, degradation and mechanical tests	[189]

Methods: for abbreviations see footnote to Table 3.

**Table 8:** Summary on covalent modification of cellulose micro- and nanomaterials by grafting “from” technique.

Type of Cellulose	Grafted Polymer	Cellulose Premodification/Polymerization Technique	Filler Content (wt%)	Matrix Aliphatic Polyester	Processing/ Design	Characterization Methods	Refs.
CNC	PLA	−/ROP	1 or 5	PLA	Extrusion + Casting/Films	Elemental analysis, FTIR, SEM, TEM, XRD, TGA	[200]
CNC	PLLA, PDLA	−/ROP	1–10	PLA	Casting/Films	NMR, FTIR, XRD, TEM, SEM, POM, DSC, mechanical tests	[201]
CNW	PLLA	−/ROP	2, 4 or 8	PLA	Molding	FTIR, AFM, DSC, DMTA	[202]
CNC	PLLA	−/ROP	5, 10 or 20	PLA, PLGA, PHB	Casting/Films	NMR, FTIR, AFM, mechanical tests	[203,204]
CNC	PLLA, PDLA	−/ROP	5 or 15	PLLA	Casting/Films	NMR, FTIR, WAXD, POM, DSC, rheological tests	[205]
CMC	OLLA	−/ROP	10, 30 or 50	PLA	Hot pressing/Films	NMR, FTIR, SEM, DSC, mechanical tests	[82]
CNF	PLA	−/ROP	2	PLA	Extrusion/ Filaments	FTIR, SEM, WVP, oxygen permeability, water adsorption and mechanical tests	[206]
CNC	PLLA, PDLA	−/ROP	0.2, 0.5 or 1	PLA	Casting/Sheets	NMR, FTIR, GPC, TEM, SEM, DSC, TGA, rheology study	[207]
CNC	PLLA	−/ROP	5	PCL	Casting/Films	FTIR, TGA, DLS, POM, mechanical and MTT-tests	[208]
CNC	PLA	−/ROP	2	PHB	Melt mixing	NMR, FTIR, DSC, XRD, TEM, XPS, SAXS	[209]
CNF	PCL	−/ROP	10	PCL	Molding/Films	FTIR, SEM, mechanical tests	[210]
CNW	PCL	−/ROP	1 or 5	PBSA	Casting/Films	FTIR, XRD, SEM, TGA, DSC	[211]
CNC	PCL	−/ROP	0.5 or 1	PHVB	Melt Blending	FTIR, FE-SEM, DSC, HSPOM, rheological and mechanical tests	[164]
CNW	PCL	−/ROP	−	−	−	SEC, XPS, FTIR, contact angles measurements	[212]
CNW	PCL	−/ROP	0–40	PCL	Casting/Films	FTIR, TOF-SIMS, WAXS, XPS, TEM, TGA, DMA, DSC, wettability and tensile tests	[67]
CNC	PLA	APTES + 3,5-diaminobenzoic acid/ROP	1–7	PLA	Electrospining/Nanofibers film	FTIR, XPS, SEM, TGA, tensile and shape memory tests	[213]
CNC	PBS	−/Polycondensation of 1,4-butanediol and succinic anhydride	0.5, 1 or 2	PLA/PBS	Compression molding/Sheets	NMR, FTIR, XPC, SEC, elemental analysis, SEM, DSC, TGA, WAXD, DMA,	[214]
Cellulose fibers	PBA, PEHA or PMMA	Adsorption of monomers/radical polymerization	50	PLA	Hot pressing/Sheets	Optical microscopy, SEM, DMA, fungal growth test	[215]
CNC	PBMA	α-bromoisobutyryl bromide/ATRP	0.5, 1 or 3	PCL	Extrusion + hot pressing/Sheets	FTIR, AFM, SEM, wettability, mechanical tests	[216]
CNF	PMMA	Oxidation with TEMPO + GPTMS/radical polymerization	1, 2 or 3	PLA	Compression molding/Sheets	FTIR, NMR, TEM, SEM, TGA, DSC, wettability and transparency study, mechanical tests	[217]
CMF	PMMA	VTES, MPTMS	2	PHB	Molding/Films	FTIR, TGA, DSC, POM, SEM, DMA and tensile tests	[218]

Methods: TOF-SIMS: time of flight secondary ion mass spectrometry; SEC: size-exclusion chromatography; SAXS: small-angle X-ray scattering; for other abbreviations see footnote to Table 3. Abbreviations: ROP: ring-opening polymerization; ATRP: atom-transfer radical polymerization; OLLA: oligo(lactic acid); PBSA: poly[(butylene succinate)-co-adipate]; PBA: poly(butyl acrylate); PEHA: poly(ethylhexyl acrylate); PMMA: poly(methyl methacrylate); PBMA: poly (butyl methacrylate); GPTMS: 3-glycidoxypropyl trimethoxysilane.

**Table 10:** Selected studies on the tensile properties of aliphatic polyester/cellulose composites.

Aliphatic Polyester	Filler	Filler Content (wt%)	Processing	Mean Tensile Modulus (GPa)		Mean Tensile Strength (MPa)		Mean Elongation at Break (%)		Refs.
				Polyester	Composite	Polyester	Composite	Polyester	Composite	
PLA	Acetylated CNF	10	Solution casting	1.08	2.37	28.3	44.1	29.9	30.1	[151]
PLA	Acetylated CNC	3	Solution casting	1.8	1.8	57	52	3.3	4.2	[140]
PLA/PBS (70/30)	CNF-fatty acids	5	Moulding	1.5	2.0	34	47	N/A	N/A	[42]
PLA	CNF-oleate	8	Solution casting	0.58	1.0	10	18.5	7	7.5	[136]
PLA	CNF-propionate	4	Solution casting	1.24	1.74	46.1	53	1.54	1.31	[152]
PLA	Silanized CNF	2	Solution casting	1.78	1.82	52.5	54.7	7.3	5.3	[177]
PLA	CNC with adsorbed PVA	1	Solution casting	1.61	1.82	47.9	45.3	3.4	12.3	[126]
PCL	CNC with adsorbed PVP	5	Solution casting	0.18	0.29	20.6	10.4	903	15	[127,131]
PLA	CNC-g-PDLA	2	Solution casting	2.50	3.25	60	80	7.5	4.5	[201]
PCL	CNC-g-PLLA	5	Solution casting	0.32	0.51	25	13	830	25	[208]
PLGA	CNC-g-PLLA	5	Solution casting	1.4	1.2	40	29	4	5	[203]
PCL	CNC-g-PCL	20	Solution casting	0.23	0.48	21	18	640	30	[67]
PLA	CNC-g-PLA	5	Electro-spinning	N/A	N/A	4.7	13	14.5	32.5	[213]
PLA	CNC-g-APTES-PEG	2	Hot pressing	N/A	N/A	25	56	1.9	3.9	[227]

## Data Availability

Not applicable.
